# The Barcelona Injury Surveillance System (BISS) for Safer Cities: Observational, Descriptive Study Using Routine Health and Police Information Databases

**DOI:** 10.2196/82079

**Published:** 2026-07-24

**Authors:** Katherine Pérez, Elena Santamariña-Rubio, Mònica Cortés-Albaladejo, Lucía Artazcoz, Adnan A Hyder, Carme Borrell

**Affiliations:** 1 Agència de Salut Pública de Barcelona (ASPB), Pl Lesseps, 1, Barcelona, 08023, Spain, 34 932027788; 2 CIBER Epidemiología y Salud Pública (CIBERESP), Madrid, Spain; 3 Institut de Recerca de Sant Pau (IR SANT PAU), Barcelona, Spain; 4 Department of Experimental and Health Sciences, Universitat Pompeu Fabra, Barcelona, Spain; 5 School of Public Health, Boston University, Boston, MA, United States

**Keywords:** injury, surveillance, monitoring, inequalities, evaluation, injury severity

## Abstract

**Background:**

Injuries are a major cause of death and disability, but cities often lack surveillance systems that can monitor injury burden across mechanisms, severity levels, and population groups. In Spain, no comprehensive city-level injury surveillance system routinely captures the full spectrum of injuries. The Barcelona Injury Surveillance System (BISS) was developed to address this gap by integrating routine health and police data.

**Objective:**

This study aims to describe the BISS, including its scope, data sources, and public health rationale, and illustrate its utility through the analysis of recent injury data in Barcelona.

**Methods:**

We conducted a descriptive study using routinely collected emergency department, hospital discharge, mortality, and police data integrated into the BISS. We analyzed nonfatal injuries in 2024, fatal injuries in 2023, and trends from 2018 onward. Injury indicators were examined by sex, age, mechanism, type, and severity. Crude and age-adjusted rates per 100,000 residents were calculated.

**Results:**

In 2024, BISS recorded 123,420 emergency department injury episodes and 18,749 injury-related hospitalizations; among residents, these figures were 99,379 and 14,319, respectively. In 2023, 695 injury-related deaths were recorded among residents. Nonfatal injuries were slightly more frequent in females, especially at older ages, whereas injury-related mortality was higher in males. Falls were the leading specified mechanism and were concentrated among older females, particularly those aged ≥75 years. Self-harm hospitalization rates were highest among females aged 15 years to 24 years, whereas self-harm mortality was higher in males. Road traffic injury and overall mortality rates were also higher in males. From 2018 to 2024, most nonfatal injury indicators increased after the decline observed in 2020, while road traffic injuries declined overall.

**Conclusions:**

BISS demonstrates the value of integrating routine health and police data to generate actionable urban injury intelligence. The findings highlight priorities for prevention, particularly falls in older females, self-harm in young females, and the persistently higher fatal injury burden among males. Integrated city-level surveillance systems such as BISS can support monitoring, equity-oriented prevention, and data-informed public health policy.

## Introduction

Injuries are a leading cause of death and disability worldwide and remain a major public health challenge across the life course [1,2]. Beyond fatalities, millions of nonfatal injuries require care in emergency departments (EDs), hospitals, and other health services, often resulting in long-term physical, psychological, and social consequences [2]. Injury burden is not evenly distributed: Marked differences are observed by age, sex, and socioeconomic status as well as by injury mechanism. Young men are disproportionately affected by road traffic injuries and interpersonal violence, whereas falls account for a substantial and increasing burden among older adults, particularly women [1,2]. These patterns underscore that injuries are not random events but largely predictable and preventable outcomes shaped by social, environmental, and policy contexts [3].

Effective prevention requires timely, valid, and policy-relevant information on the frequency, characteristics, mechanisms, and severity of injuries as well as on the population groups most affected. Public health surveillance has been defined as the continuous and systematic collection, analysis, and interpretation of health-related data for the planning, implementation, and evaluation of public health practice [4]. Applied to injuries, surveillance systems can support prevention by identifying priorities, monitoring trends, detecting inequalities, and informing the design and evaluation of interventions [5,6]. Because injury risks and consequences differ by sex and intersect with other axes of inequality, injury surveillance should also support an equity-oriented perspective [5,6].

International organizations have long emphasized the importance of standardized injury surveillance. The World Health Organization (WHO) and the US Centers for Disease Control and Prevention (CDC) developed guidance on the establishment, coding, and use of injury surveillance systems [6,7]. In Europe, injury surveillance has also been promoted through harmonized initiatives such as the European Injury Database, which supports the production of comparable indicators across countries [8-10]. Together, these initiatives have highlighted the value of routine, standardized data from health services for understanding injury burden and guiding prevention policies.

In Spain, however, there is no comprehensive injury surveillance system routinely monitoring the full spectrum of injuries at the national, regional, or city level. Existing systems have been limited in scope, with the most established example being the Barcelona Traffic Injury Information System, which has monitored road traffic injuries using multiple sources since the late 1990s [11], or fatal injuries or injury discharges at the national level reported via Eurostat [10]. At the same time, other routine data sources—such as ED records, hospital discharge data, mortality registers, and police records—offer an important but underused opportunity to build broader injury surveillance capacity particularly at the local level, where prevention can be implemented. Integrating these sources is essential to moving beyond a fragmented understanding of injuries and toward a more complete picture of their magnitude, mechanisms, severity, and social patterning.

The Barcelona Injury Surveillance System (BISS) was developed by the Public Health Agency of Barcelona to address this gap. BISS brings together existing routine health and police data to support the epidemiological surveillance of injuries in the city, including road traffic injuries, falls, violence, self-harm, and other external causes treated in health services or recorded in mortality data. By generating standardized indicators and enabling analyses by sex, age, injury mechanism, severity, and other axes of inequality, BISS aims to support priority setting, prevention planning, monitoring, and evaluation from an urban and equity-oriented public health perspective.

This study aimed to describe the BISS, including its scope, data sources, and public health rationale, and illustrate its utility through the analysis of recent injury data in Barcelona.

## Methods

### Study Design

This observational, descriptive study of injury epidemiology used routinely collected health data in Barcelona integrated into a city-wide injury surveillance system. The BISS compiles and analyzes data on injuries from multiple existing databases (health, administrative, and police records) in a continuous manner. The study design was cross-sectional and retrospective, using pre-existing data. Key elements of the design include the integration of an established road traffic injury registry (the Barcelona Traffic Injury Information System, now a subsystem called BISS-RTI for road traffic injuries) into a broader all-injury surveillance framework. The analysis presented focused on injury data from the year 2024 for emergencies and hospitalizations, 2023 for mortality, and 2018 onward for trends.

### Setting

The study was set in the city of Barcelona, Spain, an urban area with approximately 1.6 million residents within a larger metropolitan region. The BISS covers injuries occurring among all persons in Barcelona, including residents, workers, visitors, and commuters within the city. We report on injuries that occurred or were treated in Barcelona during 2024 (with trend analyses from 2018 onward), encompassing incidents across the entire city.

### Participants and Study Population

The BISS does not enroll participants directly but instead includes all injury cases meeting specific criteria from each data source. The target population for surveillance is everyone who lives, works, or spends time in Barcelona—effectively the entire population at risk in the city. We included injury episodes (events) rather than unique individuals because one person may suffer multiple injuries over time.

**Figure 1.**
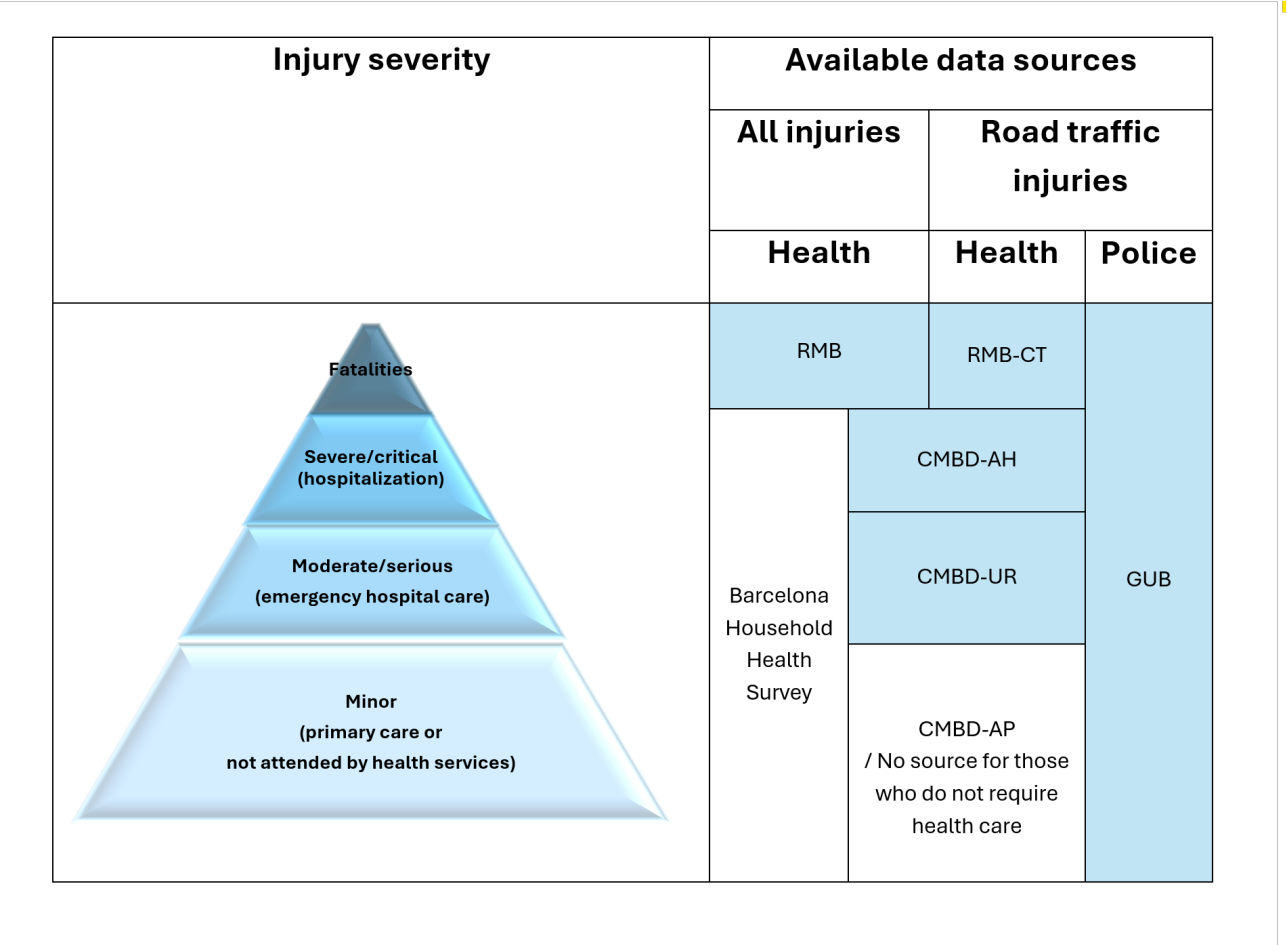
Pyramidal distribution of injuries due to external causes according to severity and available data sources for all injuries and specifically for traffic collision injuries; blue shading shows the coverage of the Barcelona Injury Surveillance System (BISS). CMBD: Register of the Minimum Basic Data Set in Spain; CMBD-AH: CMBD of hospital discharges; CMBD-AP: CMBD of primary care visits; CMBD-UR: CMBD of hospital emergency department visits; GUB: Register of Accidents and Victims by the Barcelona City Police, which does not include information on the nature and severity of injuries; RMB: Barcelona Mortality Register; RMB-CT: Barcelona Traffic Collision Mortality Register.

We provide a visual overview of the data and inclusion process: Figure 1 illustrates the “injury pyramid” for Barcelona, showing all levels of injury severity, the available data sources at each level, and the portion covered by BISS multiple (not linked) databases (shaded in the figure). At the base, there are minor injuries, which are the most common and are either treated in primary care or do not require medical attention. The next level consists of moderate to severe injuries, which are typically treated in EDs. Serious or critical injuries, although less common, require emergency medical attention and frequently lead to hospital admission for further treatment. At the top of the pyramid are fatal injuries, some of which are treated in health services (emergency care and hospitalization), while others result in death at the site of the injury before medical help can be provided.

BISS covers moderate to fatal injuries by leveraging existing routine data sources in Barcelona, primarily health-related records that document injury diagnoses and mechanisms but also health surveys (no data included here). It includes moderate to critical injuries due to external causes using data from the Register of the Minimum Basic Data Set in Spain (CMBD; acronym in Catalan) for hospital emergencies and discharges. Additionally, fatalities resulting from external causes among Barcelona residents are recorded through the Barcelona Mortality Register (RMB; acronym in Catalan). Currently, milder injuries are not included, despite the availability of CMBD data for primary care, as the quality of injury coding from this source has not yet been assessed. However, this may be considered in future phases of the system.

In the specific case of BISS-RTI, in addition to moderate to critical injuries from the CMBD of emergencies and hospital discharges, there are 2 sources for traffic injuries: the Barcelona Traffic Collision Mortality Register (RMB-CT; acronym in Catalan), which provides information on all individuals who have died from traffic-related incidents in Barcelona including injury diagnoses, and the Road Traffic Collisions Register from the Municipal Police (GUB; acronym in Catalan), which is an exhaustive record of all traffic collisions in Barcelona and the individuals injured, ranging from minor to fatal. Although the GUB does not include injury diagnoses, it provides detailed information on the circumstances of each collision.

Eligibility criteria were defined by injury mechanism and severity using diagnostic codes and external cause codes. In general, BISS covers injuries requiring medical attendance up to fatalities, while minor injuries not requiring medical attention (or only primary care) are not yet captured. Specifically, to be included in BISS analyses, a case had to satisfy the data inclusion criteria for one of the BISS databases as outlined in Table 1. Table 1 also details the algorithms and codes used to identify eligible injury records from each source, such as *International Classification of Diseases, Tenth Revision, Clinical Modification (ICD-10-CM*) external cause codes indicating injuries due to external causes, and location filters (eg, resident of Barcelona for mortality data). When there was an injury-related fatality at the ED or during hospitalization, records were classified according to the outcome (fatal vs nonfatal) to avoid double-counting deaths. In contrast, ED and hospitalization databases were not linked at the person level due to the lack of a personal identifier; consequently, some cases initially treated in the ED and later hospitalized may be counted in both sources.

**Table 1.** Barcelona Injury Surveillance System (BISS) databases and their sources with the inclusion criteria as well as injury subset selection and defined indicators for monitoring each injury subset.

Data source	Database inclusion criteria	Database	Definition criteria for the injury subset	Injury subset	Indicators
CMBD_UR^a^	Hospital in the city of Barcelona, regardless of the municipality of residence of the injured person Any of the following *ICD-10-CM^b^* codes in any of the diagnostic fields: S00-S99, T07, T14, T30-T32, T15-T28, T33-T34, T51-T76, T79, T36-T50 with a 6th digit equal to 1, 2, 3, or 4 (from codes T36.9, T37.9, T39.9, T41.4, T42.7, T43.9, T45.9, T47.9, and T49.9, including those in the 5th digit with 1, 2, 3, or 4), ending with the letter A, B, or C (“initial contact”) or without a letter OR any of the following *ICD-10-CM* codes in any of the external cause fields: V00-V99, W00-X58, X71-X83, X92-Y09, Y21-Y33, Y35-Y38 Assistance in hospital emergency departments	ED^c^	Exclude deaths	A_Episodes of nonfatal injuries treated in the hospital emergency departments of Barcelona, including residents and nonresidents of Barcelona	1 Nonfatal Emergency Department Visits for All Injuries 2 Nonfatal Emergency Department Visits for Severe Injuries 3 Nonfatal Assault-Related Emergency Department Visits 4 Nonfatal Intentional Self-Harm Emergency Department Visits 5 Nonfatal Unintentional Fall-Related Injury Emergency Department Visits 6 Nonfatal Unintentional Motor Vehicle Traffic-Related Emergency Department Visits 7 Nonfatal Unintentional Fire-Related Emergency Department Visits 8 Nonfatal Unintentional Nondrug Poisoning Emergency Department Visits 9 Nonfatal Unintentional Drug Poisoning Emergency Department Visits 10 Nonfatal TBI^d^ Emergency Department Visits
CMBD_UR	Hospital in the city of Barcelona, regardless of the municipality of residence of the injured person Any of the following *ICD-10-CM* codes in any of the diagnostic fields: S00-S99, T07, T14, T30-T32, T15-T28, T33-T34, T51-T76, T79, T36-T50 with a 6th digit equal to 1, 2, 3, or 4 (from codes T36.9, T37.9, T39.9, T41.4, T42.7, T43.9, T45.9, T47.9, and T49.9, including those in the 5th digit with 1, 2, 3, or 4), ending with the letter A, B, or C (“initial contact”) or without a letter OR any of the following *ICD-10-CM* codes in any of the external cause fields: V00-V99, W00-X58, X71-X83, X92-Y09, Y21-Y33, Y35-Y38 Assistance in hospital emergency departments	ED	Any of the following codes in any of the external cause fields: *ICD-10-CM* codes for road traffic injuries: V00.0, V00.1, V00.8, except V00.81, V00.82; V01-V06.1/.9; V09.2/.3/.9, V10-V18/V20-V28.3/.4/.5/.9; V19/V29/V39/V49/V59/V69/V79.4/.5/.6/.9; V30-V38/V40-V48/V50-V58/V60-V68/V70-V78.4/.5/.6/.7/.9; V81.1, V82.1/.9, V83-V86.0/.1/.2/.3/.4, V87, V89.2/.3/.9 ending with the letter A, B, or C (“initial contact”) or without a letter OR an economic financing regime that appears in the CMBD as mutual society for traffic accidents Exclude deaths	B_Episodes of nonfatal injuries due to traffic collisions treated in the hospital emergency departments of Barcelona, including residents and nonresidents of Barcelona	23 Nonfatal Emergency Department Visits for Road Traffic–Related Injuries 24 Nonfatal Emergency Department Visits for Road Traffic–Related Severe Injuries 25 Nonfatal Emergency Department Visits for Road Traffic–Related Pedestrian Injuries 26 Nonfatal Emergency Department Visits for Road Traffic–Related Pedestrian Transport User Injuries 27 Nonfatal Emergency Department Visits for Road Traffic–Related Cyclist Injuries 28 Nonfatal Emergency Department Visits for Road Traffic–Related Motorcyclist Injuries 29 Nonfatal Emergency Department Visits for Road Traffic–Related Car User Injuries 30 Nonfatal Emergency Department Visits for Road Traffic–Related TBI
CMBD_AH^e^	Hospital in the city of Barcelona, regardless of the municipality of residence of the injured person Any of the following *ICD-10-CM* codes in any of the diagnostic fields: S00-S99, T07, T14, T30-T32, T15-T28, T33-T34, T51-T76, T79, T36-T50 with 6th digit equal to 1, 2, 3, or 4 (of the codes T36.9, T37.9, T39.9, T41.4, T42.7, T43.9, T45.9, T47.9, and T49.9, those in which the 5th digit is 1, 2, 3, or 4), ending with the letter A, B, or C (“initial contact”) or without a letter Urgent health care (unscheduled)	HOSP^f^	Exclude deaths	C_Episodes of nonfatal injury discharged by admission to a hospital in Barcelona, including residents and nonresidents of Barcelona	11 Nonfatal Hospitalizations for All Injuries 12 Nonfatal Hospitalizations for Severe Injuries 13 Nonfatal Assault-Related Hospitalizations 14 Nonfatal Intentional Self-Harm Hospitalizations 15 Nonfatal Unintentional Fall-Related Injury Hospitalization 16 Nonfatal Unintentional Motor Vehicle Traffic-Related Hospitalizations 17 Nonfatal Unintentional Fire-Related Injury Hospitalizations 18 Nonfatal Unintentional Nondrug Poisoning Hospitalizations 19 Nonfatal Unintentional Drug Poisoning Hospitalizations 20 Nonfatal TBI Hospitalizations 21 Nonfatal Hospitalizations for Long Bone Fracture 22 Nonfatal Fall-Related Hip Fracture Hospitalizations in Persons ≥65 Years Old
CMBD_AH	Hospital in the city of Barcelona, regardless of the municipality of residence of the injured person Any of the following *ICD-10-CM* codes in any of the diagnostic fields: S00-S99, T07, T14, T30-T32, T15-T28, T33-T34, T51-T76, T79, T36-T50 with 6th digit equal to 1, 2, 3, or 4 (of the codes T36.9, T37.9, T39.9, T41.4, T42.7, T43.9, T45.9, T47.9, and T49.9, those in which the 5th digit is 1, 2, 3, or 4), ending with the letter A, B, or C (“initial contact”) or without a letter Urgent health care (unscheduled)	HOSP	Any of the following codes in any of the external cause fields: *ICD-10-CM* codes for road traffic injuries: V00.0, V00.1, V00.8, except V00.81, V00.82; V01-V06.1/.9; V09.2/.3/.9, V10-V18/V20-V28.3/.4/.5/.9; V19/V29/V39/V49/V59/V69/V79.4/.5/.6/.9; V30-V38/V40-V48/V50-V58/V60-V68/V70-V78.4/.5/.6/.7/.9; V81.1, V82.1/.9, V83-V86.0/.1/.2/.3/.4, V87, V89.2/.3/.9 ending with the letter A, B, or C (“initial contact”) or without a letter OR an economic financing regime that appears in the CMBD as mutual society for traffic accidents Exclude deaths	D_Episodes of nonfatal injuries due to traffic collisions discharged due to admission to a hospital in Barcelona, including residents and nonresidents of Barcelona	31 Nonfatal Hospitalizations for Road Traffic–Related Injuries 32 Nonfatal Hospitalizations for Road Traffic–Related Severe Injuries 33 Nonfatal Hospitalizations for Road Traffic–Related Pedestrian Injuries 34 Nonfatal Hospitalizations for Road Traffic–Related Pedestrian Transport User Injuries 35 Nonfatal Hospitalizations for Road Traffic–Related Cyclist Injuries 36 Nonfatal Hospitalizations for Road Traffic–Related Motorcyclist Injuries 37 Nonfatal Hospitalizations for Road Traffic–Related Car User Injuries 38 Nonfatal Hospitalizations for Road Traffic–Related TBI
RMB^g^	Any of the following *ICD-10* codes in the underlying cause of death field: V01–Y36, Y85–Y87, Y89, U01–U03 to the CBD^h^	MORTALITY^i^	Municipality of death: Barcelona	E_People residing in Barcelona who died from injuries due to external causes in Barcelona	39 Injury Fatalities 40 Unintentional Drowning Fatalities 41 Unintentional Fall-Related Fatalities 42 Unintentional Fire-Related Fatalities 43 Unintentional Motor Vehicle Traffic Fatalities 44 Unintentional Nondrug Poisoning Fatalities 45 Unintentional Drug Overdose Fatalities 46 Unintentional Suffocation Fatalities 47 Suicides 48 Homicides
RMB_CT^j^	Death in Barcelona due to traffic collision	MORTALITY_RTI^k^	Place of collision: Barcelona	F_Residents and nonresidents of Barcelona who died in Barcelona due to road traffic injuries that occurred in Barcelona	49 Road Traffic–Related Injury Fatalities 50 Pedestrian Road Traffic–Related Injury Fatalities 51 Cyclist Road Traffic–Related Injury Fatalities 52 Motorcyclist Road Traffic–Related Injury Fatalities 53 TBI Road Traffic–Related Injury Fatalities
GUB^l^	People involved in a road traffic collision who have been injured according to police criteria (who have required some type of medical assistance)	INJURED_RTI^m^	For people injured in the same collision, information about the collision summarized in a single record	G_Road traffic collisions in Barcelona in which a person, resident or nonresident of Barcelona, has been injured (required some type of medical assistance)	54 Road Traffic–Related Injury Collisions
GUB	People involved in a road traffic collision who have been injured according to police criteria (who have required some type of medical assistance)	INJURED_RTI	People injured	H_Residents and nonresidents of Barcelona injured (required some type of medical assistance) due to a traffic collision in Barcelona	55 Road Traffic–Injured People 56 Road Traffic–Injured Pedestrian 57 Road Traffic–Injured Pedestrian Transport Users 58 Road Traffic–Injured Motorcyclist 59 Road Traffic–Injured Cyclist 60 Road Traffic–Injured Car User

a CMBD_UR: register of the Minimum Basic Data Set of hospital emergency department visits.

b *ICD-10-CM*: *International Classification of Diseases, Tenth Revision, Clinical Modification*.

c Emergency department visit data.

d TBI: traumatic brain injury.

e CMBD_AH: register of the Minimum Basic Data Set of hospital discharges

f Hospital discharge data.

g RMB: Barcelona Mortality Register.

h CBD: basic cause of death.

i Injury-related mortality data.

j RMB_CT: Barcelona Traffic Collision Mortality Register.

k Road traffic injury–related mortality data.

l GUB: Register of Accidents and Victims by the Barcelona City Police.

m Data for people with a road traffic–related injury.

### Data Sources

Multiple data sources were used to construct the BISS databases, each providing information on different segments of the injury pyramid. All sources consist of routinely collected administrative or medical records (and police records for road traffic injuries). The sources and their use in BISS are described in the following sections.

#### Hospital Emergency Department Data (CMBD-UR)

The Urgencies Register (CMBD-UR) is a component of the regional CMBD and captures all ED visits in hospitals and emergency centers. For BISS, we extracted all ED records with an injury-related diagnosis or external cause code representing moderate or serious nonfatal injuries that received medical treatment. Each ED record includes up to 8 *ICD-10-CM* diagnosis codes and corresponding external cause codes for the injury. These data were considered comprehensive for hospital-attended injuries in the city, as the CMBD covers all public and most private hospitals in Catalonia.

#### Hospital Discharge Data (CMBD-AH)

The Hospital Discharge Register, another part of the CMBD, records all inpatient admissions in Catalonia’s hospitals. We included records of patients hospitalized for injury (again identified via *ICD-10-CM* external cause and diagnosis codes). These represent serious-to-critical injuries requiring admission. Each record can contain up to 15 diagnosis codes. By using both ED and admission data, BISS distinguishes injuries treated and released from the hospital versus those severe enough to require hospitalization.

#### Mortality Register (RMB)

The RMB collects data on all deaths of Barcelona residents, with cause of death coded in *ICD-10*. For BISS, we extracted injury-related deaths (external cause of mortality codes V01-Y89). The RMB data ensure coverage of fatal injuries among city residents, whether the death occurred in Barcelona or elsewhere. However, the mortality database does not provide detailed injury diagnoses (beyond the underlying cause of death), which limits certain indicators (eg, we cannot identify trauma subtype for all fatalities).

#### Traffic Fatalities Register (RMB-CT)

The RMB-CT is a specialized subset of the mortality data focusing on road traffic injury–related deaths in Barcelona. It is compiled by deterministically linking multiple sources: the general mortality data, records from the Institute of Legal Medicine and Forensic Sciences of Catalonia (ie, autopsy and forensic reports), civil death certificates, and police reports. This person-level data linkage allows the collection of detailed information on each traffic fatality, including injury diagnoses from an autopsy, circumstances of the crash, and victim details. The RMB-CT thus provides rich verification of road traffic death cases beyond the death certificate alone. Data from RMB-CT feed into the BISS MORTAL-RTI database (fatal road traffic injuries). The RMB includes all deaths of individuals residing in Barcelona, regardless of whether the death occurred within the city or elsewhere (manual available at [12]).

#### Police Traffic Injury Database (GUB)

The GUB records all traffic collisions in Barcelona that result in personal injury (from minor to fatal). This source does not include medical information like injury diagnoses, but it captures the context of each collision (eg, date, location, types of vehicles, road conditions) and basic information on persons injured (age, sex, road user role). The police criteria consider a [13] person injured if they require any form of medical attention at the scene or afterward. The GUB data are used for the BISS INJURED-RTI database (nonfatal traffic injuries, including minor injuries not seen in hospital records) and for calculating total collisions and total injured persons in traffic (indicators 54‐60). Police data were not directly linked to hospital records at the individual level for this analysis (ie, we did not attempt to deduplicate police-reported injuries against hospital-treated injuries) but were rather analyzed as a complementary dataset to provide information on minor injuries and collision circumstances [14].

Therefore, the BISS-RTI is fed by the 3 data sources from the Barcelona Traffic Injuries and Accidents Information System [14-17]:

- The CMBD-AH and CMBD-UR, using an injury definition for traffic-related incidents established by the “Working Group of the Spanish Society of Epidemiology on the measurement of the health impact of injuries in Spain” [18]
- The GUB, which is based on traffic collision reports from the Barcelona City Police, providing comprehensive information on the circumstances of collisions that occur within the city
- The RMB-CT

### BISS Databases and Injury Subsets and Surveillance Indicators

Based on the data sources from the CMBD-UR, CMBD-AH, RMB, RMB-RTI, and GUB and applying the data inclusion criteria outlined in Table 1, 5 databases were generated for the BISS: ED: emergency department visit data; HOSP: hospital discharge data; MORTALITY: injury-related mortality data; MORTAL-RTI: road traffic injury–related mortality data; and INJURED-RTI: data for people with a road traffic–related injury.

From these 5 databases (ED, HOSP, MORTALITY, MORTAL-RTI, and INJURED-RTI), 8 subsets of injuries to be monitored were generated (one for general injuries and another for traffic collision injuries) and labeled as A, B, C, D, E, F, G, and H.

Table 1 presents the data sources feeding into the BISS, the 5 derived databases, and the 8 resulting injury subsets. They further list the selection criteria applied to each data source and subset, along with the corresponding surveillance indicators used for monitoring. An episode of injury refers to an incident that, due to an external cause, results in harm to an individual. For nonfatal injuries, we referred to episodes rather than individuals, as a single person may experience multiple injury episodes throughout their lifetime.

### Variables

The primary outcomes of interest were counts and rates of injury cases across various categories (nonfatal injuries treated in EDs, nonfatal injuries requiring hospitalization, and fatal injuries). Rather than traditional “exposures” and “confounders” as in an etiologic study, this surveillance system tracked descriptive variables such as demographics, injury mechanisms, and severities for each case. Key variables included in BISS databases are listed in Table 2. These include basic demographics (sex, age, and country and municipality of residence), injury descriptors (date of injury or admission, diagnosis codes, and external cause codes specifying mechanism and intent), clinical outcomes (hospital discharge disposition, such as survival or death, and injury severity), and traffic-specific variables for road traffic injuries (eg, road user type, collision circumstances from police records). Table 2 displays which variables are available in each of the 5 BISS databases: ED (ED visits), HOSP (hospital admissions/discharges), MORTALITY (fatal injuries among Barcelona residents), MORTAL-RTI (fatal road traffic injuries in Barcelona), and INJURED-RTI (police-reported traffic collision injuries). A “✓” indicates the variable is present in a given database. For example, injury diagnosis codes (*ICD-10-CM*) are recorded in the health databases (ED, HOSP) with multiple diagnosis fields available and in the forensic mortality data (MORTAL-RTI) but are not recorded in the police injury database. External cause codes (*ICD-10-CM*) for the injury mechanism are captured in ED and HOSP records but not in mortality data. The severity of injuries is determined differently in police records versus medical records: Medical records allow derivation of severity via the Maximum Abbreviated Injury Scale (MAIS; from *ICD-10-CM* diagnoses), whereas the police database (INJURED-RTI) uses its own criterion of whether an injury required medical attention (not anatomic severity score). All coding frameworks (*ICD-10-CM* for diagnoses and external causes and *ICD-10* for causes of death) and classification schemes (eg, CDC’s injury matrices for intent and mechanism) used to define variables are consistent with international standards.

Table 3 provides a description of the variables considered in the BISS, including the possible values assigned to each. A more detailed description of each variable is provided in Multimedia Appendix 1.

**Table 2.** Variables included in each of the Barcelona Injury Surveillance System (BISS) databases.

Variables	Databases				
	ED^a^	HOSP^b^	MORTALITY^c^	MORTAL-RTI^d^	INJURED-RTI^e^
Admission date	✓	✓	N/A^f^	N/A	N/A
Discharge date	✓	✓	N/A	N/A	N/A
Date of death	N/A	N/A	✓	✓	N/A
Collision date	N/A	N/A	N/A	✓	✓
Sex	✓	✓	✓	✓	✓
Age	✓	✓	✓	✓	✓
Country of origin	N/A	N/A	✓	✓	✓
Municipality of residence	✓	✓	Only includes residents of Barcelona	✓	✓
Basic primary health care area (ABS)	✓	✓	✓	✓	✓
Neighborhood of residence for Barcelona residents	N/A	N/A	✓	N/A	✓
Economic financing regime	✓	✓	N/A	N/A	N/A
Situation of the person injured at discharge	✓	✓	N/A	N/A	N/A
Diagnosis of the lesion (*ICD-10-CM^g^* codes)	✓ (8 diagnostic fields)	✓ (15 diagnostic fields)	N/A	✓ (15 diagnostic fields)	N/A
External cause (*ICD-10-CM* codes)	✓ (8 fields of external cause)	✓ (5 fields of external cause)	N/A	N/A	N/A
Basic cause of death (*ICD-10* codes)	N/A	N/A	✓	✓	N/A
Type of lesion and anatomical region affected	✓	✓	N/A	✓	N/A
Mechanism and intentionality of the injury	✓	✓	✓	N/A	N/A
Type of user	✓	✓	N/A	✓	✓
Severity	✓	✓	N/A	✓	✓^h^
Collision variables (eg, type of collision, type of road, time, use of safety accessories, type of lighting)	N/A	N/A	N/A	N/A	✓

a Emergency department visit data.

b Hospital discharge data.

c Injury-related mortality data.

d Road traffic injury–related mortality data.

e Road traffic injury data from the Register of Accidents and Victims by the Barcelona City Police (GUB).

f N/A: not applicable.

g *ICD-10-CM*: *International Classification of Diseases, Tenth Revision, Clinical Modification*.

h Severity according to police criteria, which considers that a person involved in the collision is injured if they require some type of medical attention

**Table 3.** Description and values of variables considered in the Barcelona Injury Surveillance System (BISS).

Variables	Description	Values
Sex	N/A^a^	Female, male
Age (years)	N/A	0‐14, 15‐24, 25‐44, 45‐64, 65‐74, ≥75
Country of origin	Country according to National Statistic Institute codes	N/A
Municipality of residence	N/A	Barcelona, outside Barcelona
Basic Health Area (ABS; acronym in Catalan)	Elementary territorial unit through which primary health care services are organized	67 areas
Neighborhood residence	For residents of Barcelona	73 neighborhoods
Economic financing regime	N/A	Work accident insurance, traffic collision insurance, CatSalut, and other
Situation of the person injured at discharge	N/A	Death, hospital admission, discharge at home, others
Injury diagnoses	*ICD-10-CM^b^* codes	8 fields for the ED^c^ database; 15 fields for the HOSP^d^ and MORTAL-RTI^e^ databases
External cause	*ICD-10-CM* codes	8 fields for the ED database; 5 fields for the HOSP database
Basic cause of death	*ICD-10* codes	N/A
Type of injury	Derived from the injury diagnostic *ICD-10-CM* codes and classified using the matrix proposed by the CDC^f^ [19]	N/A
Anatomical region affected	Derived from the injury diagnostic *ICD-10-CM* codes and classified using the matrix proposed by the CDC [19]	N/A
Mechanism and intentionality of fatal injuries	Derived from the Basic Cause of Death *ICD-10* codes following the CDC’s classification system [20]	N/A
User type for RTI^g^	Derived from the external cause V-codes in nonfatal RTI from the ED and HOSP databases; in contrast, a dedicated variable already included in the MORTAL-RTI and INJURED-RTI^h^ databases	Pedestrian, passenger car users, motorcyclist, cyclist, van or truck user, bus user, and other users
Severity	Derived from the MAIS^i^ (ranging from 1 to 6), calculated from the AIS^j^ (ranging from 1 to 6) of each of the valid injury codes [21]	MAIS<3; MAIS≥3
Collision variables	The INJURED-RTI database, managed by the Barcelona Urban Police, includes a wide range of variables related to traffic collisions and their circumstances, such as the type of collision, type of road, time of the incident, type of lighting, weather conditions, and the use of safety accessories by the individuals involved.	N/A

a N/A: not applicable.

b *ICD-10-CM*: *International Classification of Diseases, Tenth Revision, Clinical Modification*.

c Emergency department visits.

d Hospital discharges.

e Fatal road traffic–related injuries in Barcelona.

f CDC: Centers for Disease Control and Prevention.

g RTI: road traffic injury.

h Police-reported traffic collision injuries in Barcelona.

i MAIS: Maximum Abbreviated Injury Score.

j AIS: Abbreviated Injury Score.

### Selection and Definition of Indicators

In addition to these variables, the BISS defines a set of surveillance indicators. Each indicator corresponds to a specific subset of injuries (eg, by severity, mechanism) and a measure (number of cases, rates). For example, indicators include counts of nonfatal hospital-treated injuries, counts of traffic-related injuries, counts of severe injuries, mortality counts by cause, and population-based rates for these events. These indicators were carefully selected to ensure comparability at the international level, aligning with global standards. The system relies on standard classifications, as outlined in the previous section, to provide a consistent and reliable framework for injury surveillance and analysis.

The selection of the indicator package for the BISS was conducted through an extensive review of scientific literature and official sources. The process involved searches in PubMed, as well as portals of the Spanish Ministry of Health, the European Commission, and the US CDC. Key reference publications included the report by the Spanish Ministry of Health, *Indicators of Morbidity and Mortality of Injuries Due to Traffic Accidents* [22], and the European Core Health Indicators proposal [23].

Additionally, publications and tools from the European Injury Database of the European Commission [24] were considered, as well as the CDC’s *State Injury Indicators Report: Instructions for Preparing 2020 Data* [7] and associated tools [25]. These references provided a robust basis for selecting internationally aligned and evidence-based indicators.

The BISS compiles a comprehensive set of 60 indicators organized into 5 categories to monitor the morbidity and mortality associated with injuries from external causes and to track their temporal evolution (Table 4):

1. Nonfatal injury indicators (indicators 1‐22): These indicators track nonfatal injury episodes based on the type of health care required (eg, hospital emergency or hospitalization departments), the mechanism and intentionality of the injury, and the severity.
2. Traffic collision nonfatal injury indicators (indicators 23‐38): This subset includes 16 indicators specific to nonfatal injuries resulting from traffic collisions and classified by health care requirements (eg, hospital emergency or hospitalization), severity, and user type (eg, pedestrian, cyclist, driver).
3. Mortality indicators for external causes (indicators 39‐48): These 10 indicators focus on mortality caused by external factors and are categorized by the mechanism of the basic cause of death.
4. Traffic collision mortality indicators (indicators 49‐53): This set includes 5 indicators specific to traffic collision–related mortality distinguished by the type of user involved.
5. Traffic collision and injury indicators (indicators 54‐60): These 7 indicators measure the total number of traffic collisions where medical assistance was required and the total number of injured persons without differentiating severity or type of assistance but classified by the type of user.

**Table 4.** Injury indicators, subset, definitions, and limitations of each indicator from the Barcelona Injury Surveillance System (BISS).

Indicator	Injury subset	Indicator-specific definition^a^	Limitations
1_Nonfatal_Emerg_All	A^b^	All episodes	Episodes of injury treated in hospitals in Barcelona are included, but it is unknown whether the incident took place in the city or outside. As an international consensus criterion, different attendances by the same person <31 days apart are considered part of the same episode. There may be assists from the same episode that are separated by >31 days and are considered different episodes. Conversely, the same episode could be considered care separated by <31 days that belong to different episodes, in case you are not well informed whether they are successive visits or due to sequelae. The CMBD-AH^c^ is a register of hospital discharges; therefore, there may be cases of people with the injury and were admitted in the year of study but who do not appear because they were registered the following year.
11_Nonfatal_Hosp_All	C^d^	All episodes	Episodes of injury treated in hospitals in Barcelona are included, but it is unknown whether the incident took place in the city or outside. As an international consensus criterion, different attendances by the same person <31 days apart are considered part of the same episode. There may be assists from the same episode that are separated by >31 days and are considered different episodes. Conversely, the same episode could be considered care separated by <31 days that belong to different episodes, in case you are not well informed whether they are successive visits or due to sequelae. The CMBD-AH is a register of hospital discharges; therefore, there may be cases of people with the injury and were admitted in the year of study but who do not appear because they were registered the following year.
2_Nonfatal_Emerg_Severe	A	Episodes with MAIS^e^ ≥3	Episodes of injury treated in hospitals in Barcelona are included, but it is unknown whether the incident took place in the city or outside. As an international consensus criterion, different attendances by the same person <31 days apart are considered part of the same episode. There may be assists from the same episode that are separated by >31 days and are considered different episodes. Conversely, the same episode could be considered care separated by <31 days that belong to different episodes, in case you are not well informed whether they are successive visits or due to sequelae. The CMBD-AH is a register of hospital discharges; therefore, there may be cases of people with the injury and were admitted in the year of study but who do not appear because they were registered the following year.
12_Nonfatal_Hosp_Severe	C	Episodes with MAIS ≥3	Episodes of injury treated in hospitals in Barcelona are included, but it is unknown whether the incident took place in the city or outside. As an international consensus criterion, different attendances by the same person <31 days apart are considered part of the same episode. There may be assists from the same episode that are separated by >31 days and are considered different episodes. Conversely, the same episode could be considered care separated by <31 days that belong to different episodes, in case you are not well informed whether they are successive visits or due to sequelae. The CMBD-AH is a register of hospital discharges; therefore, there may be cases of people with the injury and were admitted in the year of study but who do not appear because they were registered the following year.
3_Nonfatal_Emerg_Assault	A	Episodes with any of the following codes in any diagnosis or external cause field: *ICD-10-CM*^f^ codes for assault-related injuries: X92-Y09; T36-T50 with 6th character=3 (note: include T36.9, T37.9, T39.9, T41.4, T42.7, T43.9, T45.9, T47.9, and T49.9 with 5th character=3 [intent information for these codes included in the 5th character and not the 6th]); T51-T65 with 6th character=3 (note: include T51.9, T52.9, T53.9, T54.9, T56.9, T57.9, T58.0, T58.1, T58.9, T59.9, T60.9, T61.0, T61.1, T61.9, T62.9, T63.9, T64, and T65.9 with a 5th character =3 [intent information for these codes included in the 5th character and not the 6th]); T71 with 6th character=3; T74, T76, Y38	Episodes of injury treated in hospitals in Barcelona are included, but it is unknown whether the incident took place in the city or outside. As an international consensus criterion, different attendances by the same person <31 days apart are considered part of the same episode. There may be assists from the same episode that are separated by >31 days and are considered different episodes. Conversely, the same episode could be considered care separated by <31 days that belong to different episodes, in case you are not well informed whether they are successive visits or due to sequelae. The CMBD-AH is a register of hospital discharges; therefore, there may be cases of people with the injury and were admitted in the year of study but who do not appear because they were registered the following year.
13_Nonfatal_Hosp_Assault	C	Episodes with any of the following codes in any diagnosis or external cause field: *ICD-10-CM* codes for assault-related injuries: X92-Y09; T36-T50 with 6th characte=3 (note: include T36.9, T37.9, T39.9, T41.4, T42.7, T43.9, T45.9, T47.9, and T49.9 with 5th character=3 [intent information for these codes included in the 5th character and not the 6th]); T51-T65 with 6th character=3 (note: include T51.9, T52.9, T53.9, T54.9, T56.9, T57.9, T58.0, T58.1, T58.9, T59.9, T60.9, T61.0, T61.1, T61.9, T62.9, T63.9, T64, and T65.9 with a 5th character=3 [intent information for these codes included in the 5th character and not the 6th]); T71 with 6th character=3; T74, T76, Y38	isodes of injury treated in hospitals in Barcelona are included, but it is unknown whether the incident took place in the city or outside. As an international consensus criterion, different attendances by the same person <31 days apart are considered part of the same episode. There may be assists from the same episode that are separated by >31 days and are considered different episodes. Conversely, the same episode could be considered care separated by <31 days that belong to different episodes, in case you are not well informed whether they are successive visits or due to sequelae. The CMBD-AH is a register of hospital discharges; therefore, there may be cases of people with the injury and were admitted in the year of study but who do not appear because they were registered the following year.
4_Nonfatal_Emerg_Self-Harm	A	Episodes with any of the following codes in any diagnosis or external cause field: *ICD-10-CM* codes for intentional self-harm injuries: X71-X83; T36-50 with 6th character=2 (note: include T36.9, T37.9, T39.9, T41.4, T42.7, T43.9, T45.9, T47.9, and T49.9 with 5th character=2 [intent information for these codes included in the 5th character and not the 6th]); T51-T65 with 6th character=2 (note: include T51.9, T52.9, T53.9, T54.9, T56.9, T57.9, T58.0, T58.1, T58.9, T59.9, T60.9, T61.0, T61.1, T61.9, T62.9, T63.9, T64, and T65.9 with a 5th character=2 [intent information for these codes included in the 5th character and not the 6th]); T71 with 6th character=2; T14.91	Episodes of injury treated in hospitals in Barcelona are included, but it is unknown whether the incident took place in the city or outside. As an international consensus criterion, different attendances by the same person <31 days apart are considered part of the same episode. There may be assists from the same episode that are separated by >31 days and are considered different episodes. Conversely, the same episode could be considered care separated by <31 days that belong to different episodes, in case you are not well informed whether they are successive visits or due to sequelae. The CMBD-AH is a register of hospital discharges; therefore, there may be cases of people with the injury and were admitted in the year of study but who do not appear because they were registered the following year.
14_Nonfatal_Hosp_Self-Harm	C	Episodes with any of the following codes in any diagnosis or external cause field: *ICD-10-CM* codes for intentional self-harm injuries: X71-X83; T36-50 with 6th character=2 (note: include T36.9, T37.9, T39.9, T41.4, T42.7, T43.9, T45.9, T47.9, and T49.9 with 5th character =2 [intent information for these codes included in the 5th character and not the 6th]); T51-T65 with 6th character =2 (note: include T51.9, T52.9, T53.9, T54.9, T56.9, T57.9, T58.0, T58.1, T58.9, T59.9, T60.9, T61.0, T61.1, T61.9, T62.9, T63.9, T64, and T65.9 with a 5th character=2 [intent information for these codes included in the 5th character and not the 6th]); T71 with 6th character=2; T14.91	Episodes of injury treated in hospitals in Barcelona are included, but it is unknown whether the incident took place in the city or outside. As an international consensus criterion, different attendances by the same person <31 days apart are considered part of the same episode. There may be assists from the same episode that are separated by >31 days and are considered different episodes. Conversely, the same episode could be considered care separated by <31 days that belong to different episodes, in case you are not well informed whether they are successive visits or due to sequelae. The CMBD-AH is a register of hospital discharges; therefore, there may be cases of people with the injury and were admitted in the year of study but who do not appear because they were registered the following year.
5_Nonfatal_Emerg_Unint_Fall	A	Episodes with any of the following codes in external cause field: *ICD-10-CM* codes for unintentional fall-related injuries: V00.1-V00.8 if 6th character=1; W00-W15, W17, W19; W16 if 6th character=2, W16.42, W16.92; W18.1-W18.3	Episodes of injury treated in hospitals in Barcelona are included, but it is unknown whether the incident took place in the city or outside. As an international consensus criterion, different attendances f the same person <31 days apart are considered part of the same episode. There may be assists from the same episode that are separated by >31 days and are considered different episodes. Conversely, the same episode could be considered care separated by <31 days that belong to different episodes, in case you are not well informed whether they are successive visits or due to sequelae. The CMBD-AH is a register of hospital discharges; therefore, there may be cases of people with the injury and were admitted in the year of study but who do not appear because they were registered the following year.
15_Nonfatal_Hosp_Unint_Fall	C	Episodes with any of the following codes in external cause field: *ICD-10-CM* codes for unintentional fall-related injuries: V00.1-V00.8 if 6th character=1; W00-W15, W17, W19; W16 if 6th character=2, W16.42, W16.92; W18.1-W18.3	Episodes of injury treated in hospitals in Barcelona are included, but it is unknown whether the incident took place in the city or outside. As an international consensus criterion, different attendances by the same person <31 days apart are considered part of the same episode. There may be assists from the same episode that are separated by >31 days and are considered different episodes. Conversely, the same episode could be considered care separated by <31 days that belong to different episodes, in case you are not well informed whether they are successive visits or due to sequelae. The CMBD-AH is a register of hospital discharges; therefore, there may be cases of people with the injury and were admitted in the year of study but who do not appear because they were registered the following year.
6_Nonfatal_Emerg_Unint_MVT^g^	A	Episodes with any of the following codes in external cause field: *ICD-10-CM* codes for unintentional motor vehicle traffic-related injuries: V02-V04 (.1-.9), V09.2; V12-V14 (.3-.9), V19.4-V19.6; V20-V28 (.3-.9), V29.4-V29.9; V30-V39 (.4-.9); V40-V49 (.4-.9); V50-V59 (.4-.9); V60-V69 (.4-.9); V70-V79 (.4-.9); V80.3-V80.5, V81.1, V82.1, V83-V86 (.0-.3), V87.0-V87.8, V89.2	Episodes of injury treated in hospitals in Barcelona are included, but it is unknown whether the incident took place in the city or outside. As an international consensus criterion, different attendances by the same person <31 days apart are considered part of the same episode. There may be assists from the same episode that are separated by >31 days and are considered different episodes. Conversely, the same episode could be considered care separated by <31 days that belong to different episodes, in case you are not well informed whether they are successive visits or due to sequelae. The CMBD-AH is a register of hospital discharges; therefore, there may be cases of people with the injury and were admitted in the year of study but who do not appear because they were registered the following year.
16_Nonfatal_Hosp_Unint_MVT	C	Episodes with any of the following codes in external cause field. *ICD-10-CM* codes for unintentional motor vehicle traffic-related injuries: V02-V04 (.1-.9), V09.2; V12-V14 (.3-.9), V19.4-V19.6; V20-V28 (.3-.9), V29.4-V29.9; V30-V39 (.4-.9); V40-V49 (.4-.9); V50-V59 (.4-.9); V60-V69 (.4-.9); V70-V79 (.4-.9); V80.3-V80.5, V81.1, V82.1, V83-V86 (.0-.3), V87.0-V87.8, V89.2	Episodes of injury treated in hospitals in Barcelona are included, but it is unknown whether the incident took place in the city or outside. As an international consensus criterion, different attendances by the same person <31 days apart are considered part of the same episode. There may be assists from the same episode that are separated by >31 days and are considered different episodes. Conversely, the same episode could be considered care separated by <31 days that belong to different episodes, in case you are not well informed whether they are successive visits or due to sequelae. The CMBD-AH is a register of hospital discharges; therefore, there may be cases of people with the injury and were admitted in the year of study but who do not appear because they were registered the following year.
7_Nonfatal_Emerg_Unint_Fire	A	Episodes with any of the following codes in external cause field: *ICD-10-CM* codes for unintentional fire-related injuries: X00-X08	Episodes of injury treated in hospitals in Barcelona are included, but it is unknown whether the incident took place in the city or outside. As an international consensus criterion, different attendances by the same person <31 days apart are considered part of the same episode. There may be assists from the same episode that are separated by >31 days and are considered different episodes. Conversely, the same episode could be considered care separated by <31 days that belong to different episodes, in case you are not well informed whether they are successive visits or due to sequelae. The CMBD-AH is a register of hospital discharges; therefore, there may be cases of people with the injury and were admitted in the year of study but who do not appear because they were registered the following year.
17_Nonfatal_Hosp_Unint_Fire	C	Episodes with any of the following codes in external cause field: *ICD-10-CM* codes for unintentional fire-related injuries: X00-X08	Episodes of injury treated in hospitals in Barcelona are included, but it is unknown whether the incident took place in the city or outside. As an international consensus criterion, different attendances by the same person <31 days apart are considered part of the same episode. There may be assists from the same episode that are separated by >31 days and are considered different episodes. Conversely, the same episode could be considered care separated by <31 days that belong to different episodes, in case you are not well informed whether they are successive visits or due to sequelae. The CMBD-AH is a register of hospital discharges; therefore, there may be cases of people with the injury and were admitted in the year of study but who do not appear because they were registered the following year.
8_Nonfatal_Emerg_Unint_Nondrug	A	Episodes with any of the following codes in any diagnosis or external cause field: *ICD-10-CM* codes for unintentional nondrug poisonings: T51-T52 (.0-.3 and .8 with 6th character=1 and .91); T53 (.0-.7 with 6th character=1 and .91); T55 (.0, .1 with 6th character=1); T56 (.0-.7, .81, and .89 with 6th character =1 and .91); T57 (.0-.3 and .8 with 6th character=1 and .91); T58 (.01, .11, .2, and .8 with 6th character=1 and .91); T59 (.0-.7, .81, and .89 with 6th character=1 and .91); T60 (.0-.8 with 6th character=1 and .91); T61 (.01, .11, .77, .78, and .8 with 6th character=1 and .91); T62 (.0-.8 with 6th character=1 and .91); T64 (.01 and .81); T54.0 with 6th character=1; T65 (.0, .1, .21, .22, .29, .3-.6, .81, .83, and .89 with 6th character=1 and .91)	Episodes of injury treated in hospitals in Barcelona are included, but it is unknown whether the incident took place in the city or outside. As an international consensus criterion, different attendances by the same person <31 days apart are considered part of the same episode. There may be assists from the same episode that are separated by >31 days and are considered different episodes. Conversely, the same episode could be considered care separated by <31 days that belong to different episodes, in case you are not well informed whether they are successive visits or due to sequelae. The CMBD-AH is a register of hospital discharges; therefore, there may be cases of people with the injury and were admitted in the year of study but who do not appear because they were registered the following year.
18_Nonfatal_Hosp_Unint_Nondrug	C	Episodes with any of the following codes in any diagnosis or external cause field: *ICD-10-CM* codes for unintentional nondrug poisonings: T51-T52 (.0-.3 and .8 with 6th character=1 and .91); T53 (.0-.7 with 6th character=1 and .91); T55 (.0, .1 with 6th character=1); T56 (.0-.7, .81, and .89 with 6th character=1 and .91); T57 (.0-.3 and .8 with 6th character=1 and .91); T58 (.01, .11, .2, and .8 with 6th character=1 and .91); T59 (.0-.7, .81, and .89 with 6th character=1 and .91); T60 (.0-.8 with 6th character=1 and .91); T61 (.01, .11, .77, .78, and .8 with 6th character=1 and .91); T62 (.0-.8 with 6th character=1 and .91); T64 (.01 and .81); T54.0 with 6th character=1; T65 (.0, .1, .21, .22, .29, .3-.6, .81, .83, and .89 with 6th character=1 and .91)	Episodes of injury treated in hospitals in Barcelona are included, but it is unknown whether the incident took place in the city or outside. As an international consensus criterion, different attendances by the same person <31 days apart are considered part of the same episode. There may be assists from the same episode that are separated by >31 days and are considered different episodes. Conversely, the same episode could be considered care separated by <31 days that belong to different episodes, in case you are not well informed whether they are successive visits or due to sequelae. The CMBD-AH is a register of hospital discharges; therefore, there may be cases of people with the injury and were admitted in the year of study but who do not appear because they were registered the following year.
9_Nonfatal_Emerg_Unint_Drug	A	Episodes with any of the following codes in any diagnosis or external cause field: *ICD-10-CM* codes for unintentional drug poisonings: T36, T37, T47 (.0-.8 with 6th character=1 and .91); T38 (.0-.7, .80, .81, .89, .90, and .99 with 6th character=1); T39 (.09, .1, .2, .31, .39, .4, and .8 with 6th character=1 and .91); T40 (.0-.4, .41, .42, .49, .5, .60, .69, .7, .71, .72, .8, .90, and .99 with 6th character=1); T41 (.0, .1, .20, .29, .3, .41, and .5 with 6th character=1); T42 (.0-.6, .71, and .8 with 6th character=1); T43 (.01, .02, .1, .20-.22, .29, .3, .4, .50, .59, .60-.65, .69, and .8, with 6th character=1 and .91); T44 and T46 (.0-.8, .90, and .99 with 6th character=1); T45 .0-.4, .51, .52, .60-.62, .69, .7, and .8 with 6th character=1 and .91)	Episodes of injury treated in hospitals in Barcelona are included, but it is unknown whether the incident took place in the city or outside. As an international consensus criterion, different attendances by the same person <31 days apart are considered part of the same episode. There may be assists from the same episode that are separated by >31 days and are considered different episodes. Conversely, the same episode could be considered care separated by <31 days that belong to different episodes, in case you are not well informed whether they are successive visits or due to sequelae. The CMBD-AH is a register of hospital discharges; therefore, there may be cases of people with the injury and were admitted in the year of study but who do not appear because they were registered the following year.
19_Nonfatal_Hosp_Unint_Drug	C	Episodes with any of the following codes in any diagnosis or external cause field: *ICD-10-CM* codes for unintentional drug poisonings: T36, T37, T47 (.0-.8 with 6th character=1 and .91); T38 (.0-.7, .80, .81, .89, .90, and .99 with 6th character=1); T39 (.09, .1, .2, .31, .39, .4, and .8 with 6th character=1 and .91); T40 (.0-.4, .41, .42, .49, .5, .60, .69, .7, .71, .72, .8, .90, and .99 with 6th character=1); T41 (.0, .1, .20, .29, .3, .41, and .5 with 6th character=1); T42 (.0-.6, .71, and .8 with 6th character=1); T43 (.01, .02, .1, .20-.22, .29, .3, .4, .50, .59, .60-.65, .69, and .8, with 6th character=1 and .91); T44 and T46 (.0-.8, .90, and .99 with 6th character=1); T45 .0-.4, .51, .52, .60-.62, .69, .7, and .8 with 6th character=1 and .91)	• Episodes of injury treated in hospitals in Barcelona are included, but it is unknown whether the incident took place in the city or outside. As an international consensus criterion, different attendances by the same person <31 days apart are considered part of the same episode. There may be assists from the same episode that are separated by >31 days and are considered different episodes. Conversely, the same episode could be considered care separated by <31 days that belong to different episodes, in case you are not well informed whether they are successive visits or due to sequelae. The CMBD-AH is a register of hospital discharges; therefore, there may be cases of people with the injury and were admitted in the year of study but who do not appear because they were registered the following year.
10_Nonfatal_Emerg_TBI^h^	A	Episodes with any of the following codes in any diagnosis field: *ICD-10-CM* diagnosis codes for the CDC^i^ proposed TBI case definition: S02.0, S02.1; S02.80, S02.81, S02.82, S02.91; S04.02, S04.03, S04.04; S06; S07.1; T74.4	Episodes of injury treated in hospitals in Barcelona are included, but it is unknown whether the incident took place in the city or outside. As an international consensus criterion, different attendances by the same person <31 days apart are considered part of the same episode. There may be assists from the same episode that are separated by >31 days and are considered different episodes. Conversely, the same episode could be considered care separated by <31 days that belong to different episodes, in case you are not well informed whether they are successive visits or due to sequelae. The CMBD-AH is a register of hospital discharges; therefore, there may be cases of people with the injury and were admitted in the year of study but who do not appear because they were registered the following year.
20_Nonfatal_Hosp_TBI	C	Episodes with any of the following codes in any diagnosis field: *ICD-10-CM* diagnosis codes for the CDC proposed TBI case definition: S02.0, S02.1; S02.80, S02.81, S02.82, S02.91; S04.02, S04.03, S04.04; S06; S07.1; T74.4	Episodes of injury treated in hospitals in Barcelona are included, but it is unknown whether the incident took place in the city or outside. As an international consensus criterion, different attendances by the same person <31 days apart are considered part of the same episode. There may be assists from the same episode that are separated by >31 days and are considered different episodes. Conversely, the same episode could be considered care separated by <31 days that belong to different episodes, in case you are not well informed whether they are successive visits or due to sequelae. The CMBD-AH is a register of hospital discharges; therefore, there may be cases of people with the injury and were admitted in the year of study but who do not appear because they were registered the following year.
21_Nonfatal_Hosp_Long-Bone	C	Episodes with any of the following codes in any diagnosis field: *ICD-10-CM* diagnosis codes proposed for long bone fracture: S72.0- S72.9; S42.2-S42.9; S82.1-S82.9	Episodes of injury treated in hospitals in Barcelona are included, but it is unknown whether the incident took place in the city or outside. As an international consensus criterion, different attendances by the same person <31 days apart are considered part of the same episode. There may be assists from the same episode that are separated by >31 days and are considered different episodes. Conversely, the same episode could be considered care separated by <31 days that belong to different episodes, in case you are not well informed whether they are successive visits or due to sequelae. The CMBD-AH is a register of hospital discharges; therefore, there may be cases of people with the injury and were admitted in the year of study but who do not appear because they were registered the following year.
22_Nonfatal_Hosp_Fall-Hip-65	C	Episodes in people >65 years old with any of the following hip fracture codes in any diagnosis field and any of the following unintentional fall codes in any external cause field: *ICD-10-CM* codes for hip fractures: S72.0, S72.1, S72.2, M97.0; ICD-10-CM codes for unintentional fall-related injuries: V00.1-V00.8 with the 6th character=1; W00-W15, W17, W19; W16 with the 6th character=2, W16.42, W16.92; W18.1-W18.3	Episodes of injury treated in hospitals in Barcelona are included, but it is unknown whether the incident took place in the city or outside. As an international consensus criterion, different attendances by the same person <31 days apart are considered part of the same episode. There may be assists from the same episode that are separated by >31 days and are considered different episodes. Conversely, the same episode could be considered care separated by <31 days that belong to different episodes, in case you are not well informed whether they are successive visits or due to sequelae. The CMBD-AH is a register of hospital discharges; therefore, there may be cases of people with the injury and were admitted in the year of study but who do not appear because they were registered the following year.
23_Nonfatal_Emerg_RTI^j^	B^k^	All episodes	Episodes of injury treated in hospitals in Barcelona are included, but it is unknown whether the incident took place in the city or outside. As an international consensus criterion, different attendances by the same person <31 days apart are considered part of the same episode. However, there could be assists derived from the same episode that are separated in time by >31 days and are being considered different episodes. Conversely, the same episode could be considered care separated by <31 days that belong to different episodes, in case you are not well informed whether they are successive visits or due to sequelae. The CMBD-AH is a register of hospital discharges; therefore, there may be cases of people with the injury and were admitted in the year of study but who do not appear because they were registered the following year.
31_Nonfatal_Hosp_RTI	D^l^	All episodes	Episodes of injury treated in hospitals in Barcelona are included, but it is unknown whether the incident took place in the city or outside. As an international consensus criterion, different attendances by the same person <31 days apart are considered part of the same episode. However, there could be assists derived from the same episode that are separated in time by >31 days and are being considered different episodes. Conversely, the same episode could be considered care separated by <31 days that belong to different episodes, in case you are not well informed whether they are successive visits or due to sequelae. The CMBD-AH is a register of hospital discharges; therefore, there may be cases of people with the injury and were admitted in the year of study but who do not appear because they were registered the following year.
24_Nonfatal_Emerg_Severe-RTI	B	Episodes with MAIS ≥3	Episodes of injury treated in hospitals in Barcelona are included, but it is unknown whether the incident took place in the city or outside. As an international consensus criterion, different attendances by the same person <31 days apart are considered part of the same episode. However, there could be assists derived from the same episode that are separated in time by >31 days and are being considered different episodes. Conversely, the same episode could be considered care separated by <31 days that belong to different episodes, in case you are not well informed whether they are successive visits or due to sequelae. The CMBD-AH is a register of hospital discharges; therefore, there may be cases of people with the injury and were admitted in the year of study but who do not appear because they were registered the following year.
32_Nonfatal_Hosp_Severe-RTI	D	Episodes with MAIS ≥3	Episodes of injury treated in hospitals in Barcelona are included, but it is unknown whether the incident took place in the city or outside. As an international consensus criterion, different attendances by the same person <31 days apart are considered part of the same episode. However, there could be assists derived from the same episode that are separated in time by >31 days and are being considered different episodes. Conversely, the same episode could be considered care separated by <31 days that belong to different episodes, in case you are not well informed whether they are successive visits or due to sequelae. The CMBD-AH is a register of hospital discharges; therefore, there may be cases of people with the injury and were admitted in the year of study but who do not appear because they were registered the following year.
25_Nonfatal_Emerg_Pedestrian	B	Episodes with any of the following codes in any external cause field: *ICD-10-CM* codes for pedestrian RTI-related injuries: V00.0; V01-V06.1/.9; V09.2/.3/.9	Episodes of injury treated in hospitals in Barcelona are included, but it is unknown whether the incident took place in the city or outside. As an international consensus criterion, different attendances by the same person <31 days apart are considered part of the same episode. However, there could be assists derived from the same episode that are separated in time by >31 days and are being considered different episodes. Conversely, the same episode could be considered care separated by <31 days that belong to different episodes, in case you are not well informed whether they are successive visits or due to sequelae. The CMBD-AH is a register of hospital discharges; therefore, there may be cases of people with the injury and were admitted in the year of study but who do not appear because they were registered the following year.
33_Nonfatal_Hosp_Pedestrian	D	Episodes with any of the following codes in any external cause field: *ICD-10-CM* codes for pedestrian RTI-related injuries: V00.0; V01-V06.1/.9; V09.2/.3/.9	Episodes of injury treated in hospitals in Barcelona are included, but it is unknown whether the incident took place in the city or outside. As an international consensus criterion, different attendances of the same person <31 days apart are considered part of the same episode. However, there could be assists derived from the same episode that are separated in time by >31 days and are being considered different episodes. Conversely, the same episode could be considered care separated by <31 days that belong to different episodes, in case you are not well informed whether they are successive visits or due to sequelae. The CMBD-AH is a register of hospital discharges; therefore, there may be cases of people with the injury and were admitted in the year of study but who do not appear because they were registered the following year.
26_Nonfatal_Emerg_PedesTransp	B	Episodes with any of the following codes in external cause field: *ICD-10-CM* codes for pedestrian transport RTI-related injuries: V00.1; V00.8, except V00.81, V00.82	Episodes of injury treated in hospitals in Barcelona are included, but it is unknown whether the incident took place in the city or outside. As an international consensus criterion, different attendances of the same person <31 days apart are considered part of the same episode. However, there could be assists derived from the same episode that are separated in time by >31 days and are being considered different episodes. Conversely, the same episode could be considered care separated by <31 days that belong to different episodes, in case you are not well informed whether they are successive visits or due to sequelae. The CMBD-AH is a register of hospital discharges; therefore, there may be cases of people with the injury and were admitted in the year of study but who do not appear because they were registered the following year.
34_Nonfatal_Hosp_PedesTransp	D	Episodes with any of the following codes in external cause field: *ICD-10-CM* codes for pedestrian transport RTI-related injuries: V00.1; V00.8, except V00.81, V00.82	Episodes of injury treated in hospitals in Barcelona are included, but it is unknown whether the incident took place in the city or outside. As an international consensus criterion, different attendances by the same person <31 days apart are considered part of the same episode. However, there could be assists derived from the same episode that are separated in time by >31 days and are being considered different episodes. Conversely, the same episode could be considered care separated by <31 days that belong to different episodes, in case you are not well informed whether they are successive visits or due to sequelae. The CMBD-AH is a register of hospital discharges; therefore, there may be cases of people with the injury and were admitted in the year of study but who do not appear because they were registered the following year.
27_Nonfatal_Emerg_Cyclist	B	Episodes with any of the following codes in external cause field: *ICD-10-CM* codes for cyclist RTI-related injuries: V10-V18.3/.4/.5/.9; V19.4/.5/.6/.9	Episodes of injury treated in hospitals in Barcelona are included, but it is unknown whether the incident took place in the city or outside. As an international consensus criterion, different attendances by the same person <31 days apart are considered part of the same episode. However, there could be assists derived from the same episode that are separated in time by >31 days and are being considered different episodes. Conversely, the same episode could be considered care separated by <31 days that belong to different episodes, in case you are not well informed whether they are successive visits or due to sequelae. The CMBD-AH is a register of hospital discharges; therefore, there may be cases of people with the injury and were admitted in the year of study but who do not appear because they were registered the following year.
35_Nonfatal_Hosp_Cyclist	D	Episodes with any of the following codes in external cause field: *ICD-10-CM* codes for cyclist RTI-related injuries: V10-V18.3/.4/.5/.9; V19.4/.5/.6/.9	Episodes of injury treated in hospitals in Barcelona are included, but it is unknown whether the incident took place in the city or outside. As an international consensus criterion, different attendances by the same person <31 days apart are considered part of the same episode. However, there could be assists derived from the same episode that are separated in time by >31 days and are being considered different episodes. Conversely, the same episode could be considered care separated by <31 days that belong to different episodes, in case you are not well informed whether they are successive visits or due to sequelae. The CMBD-AH is a register of hospital discharges; therefore, there may be cases of people with the injury and were admitted in the year of study but who do not appear because they were registered the following year.
28_Nonfatal_Emerg_Motorcyclist	B	Episodes with any of the following codes in any external cause field: *ICD-10-CM* codes for motorcyclist RTI-related injuries: V20-V28.3/.4/.5/.9; V30-V38.4/.5/.6/.7/.9; V29-V39.4/.5/.6/.9; V39	Episodes of injury treated in hospitals in Barcelona are included, but it is unknown whether the incident took place in the city or outside. As an international consensus criterion, different attendances of the same person <31 days apart are considered part of the same episode. However, there could be assists derived from the same episode that are separated in time by >31 days and are being considered different episodes. Conversely, the same episode could be considered care separated by <31 days that belong to different episodes, in case you are not well informed whether they are successive visits or due to sequelae. The CMBD-AH is a register of hospital discharges; therefore, there may be cases of people with the injury and were admitted in the year of study but who do not appear because they were registered the following year.
36_Nonfatal_Hosp_Motorcyclist	D	Episodes with any of the following codes in any external cause field: *ICD-10-CM* codes for motorcyclist RTI-related injuries: V20-V28.3/.4/.5/.9; V30-V38.4/.5/.6/.7/.9; V29-V39.4/.5/.6/.9; V39	Episodes of injury treated in hospitals in Barcelona are included, but it is unknown whether the incident took place in the city or outside. As an international consensus criterion, different attendances by the same person <31 days apart are considered part of the same episode. However, there could be assists derived from the same episode that are separated in time by >31 days and are being considered different episodes. Conversely, the same episode could be considered care separated by <31 days that belong to different episodes, in case you are not well informed whether they are successive visits or due to sequelae. The CMBD-AH is a register of hospital discharges; therefore, there may be cases of people with the injury and were admitted in the year of study but who do not appear because they were registered the following year.
29_Nonfatal_Emerg_CarUser	B	Episodes with any of the following codes in any external cause field: *ICD-10-CM* codes for car user RTI-related injuries: V40-48.4/.5/.6/.7/.9; V49.4/.5/.6/.9	Episodes of injury treated in hospitals in Barcelona are included, but it is unknown whether the incident took place in the city or outside. As an international consensus criterion, different attendances by the same person <31 days apart are considered part of the same episode. However, there could be assists derived from the same episode that are separated in time by >31 days and are being considered different episodes. Conversely, the same episode could be considered care separated by <31 days that belong to different episodes, in case you are not well informed whether they are successive visits or due to sequelae. The CMBD-AH is a register of hospital discharges; therefore, there may be cases of people with the injury and were admitted in the year of study but who do not appear because they were registered the following year.
37_Nonfatal_Hosp_CarUser	D	Episodes with any of the following codes in any external cause field: *ICD-10-CM* codes for car user RTI-related injuries: V40-48.4/.5/.6/.7/.9; V49.4/.5/.6/.9	Episodes of injury treated in hospitals in Barcelona are included, but it is unknown whether the incident took place in the city or outside. As an international consensus criterion, different attendances by the same person <31 days apart are considered part of the same episode. However, there could be assists derived from the same episode that are separated in time by >31 days and are being considered different episodes. Conversely, the same episode could be considered care separated by <31 days that belong to different episodes, in case you are not well informed whether they are successive visits or due to sequelae. The CMBD-AH is a register of hospital discharges; therefore, there may be cases of people with the injury and were admitted in the year of study but who do not appear because they were registered the following year.
30_Nonfatal_Emerg_TBI-RTI	B	Episodes with any of the following codes in any diagnosis field: *ICD-10-CM* codes proposed for TBIs: S02.0, S02.1; S02.80, S02.81, S02.82, S02.91; S04.02, S04.03, S04.04; S06; S07.1; T74.4	Episodes of injury treated in hospitals in Barcelona are included, but it is unknown whether the incident took place in the city or outside. As an international consensus criterion, different attendances by the same person <31 days apart are considered part of the same episode. However, there could be assists derived from the same episode that are separated in time by >31 days and are being considered different episodes. Conversely, the same episode could be considered care separated by <31 days that belong to different episodes, in case you are not well informed whether they are successive visits or due to sequelae. The CMBD-AH is a register of hospital discharges; therefore, there may be cases of people with the injury and were admitted in the year of study but who do not appear because they were registered the following year.
38_Nonfatal_Hosp_TBI-RTI	D	Episodes with any of the following codes in any diagnosis field: *ICD-10-CM* codes proposed for TBIs: S02.0, S02.1; S02.80, S02.81, S02.82, S02.91; S04.02, S04.03, S04.04; S06; S07.1; T74.4	Episodes of injury treated in hospitals in Barcelona are included, but it is unknown whether the incident took place in the city or outside. As an international consensus criterion, different attendances by the same person <31 days apart are considered part of the same episode. However, there could be assists derived from the same episode that are separated in time by >31 days and are being considered different episodes. Conversely, the same episode could be considered care separated by <31 days that belong to different episodes, in case you are not well informed whether they are successive visits or due to sequelae. The CMBD-AH is a register of hospital discharges; therefore, there may be cases of people with the injury and were admitted in the year of study but who do not appear because they were registered the following year.
39_Fatal_All	E^m^	All deaths	Only the deaths of people residing in Barcelona are available. People who died in Barcelona are selected regardless of where the episode of injury took place, since this information is not available. The RMB^n^ does not have information on injuries, so it is not possible to define mortality indicators based on diagnostic codes, such as TBI fatalities.
40_Fatal_Unintent-Drowning	E	Deaths with any of the following codes in the underlying cause of death field: *ICD-10* codes for unintentional drowning–related deaths: W65-W74	Only the deaths of people residing in Barcelona are available. People who died in Barcelona are selected regardless of where the episode of injury took place, since this information is not available. The RMB does not have information on injuries, so it is not possible to define mortality indicators based on diagnostic codes, such as TBI fatalities.
41_Fatal_Unintent-Fall	E	Deaths with any of the following codes in the underlying cause of death field: *ICD-10* codes for unintentional fall–related deaths: W00-W19	Only the deaths of people residing in Barcelona are available. People who died in Barcelona are selected regardless of where the episode of injury took place, since this information is not available. The RMB does not have information on injuries, so it is not possible to define mortality indicators based on diagnostic codes, such as TBI fatalities.
42_Fatal_Unintent-Fire	E	Deaths with any of the following codes in the underlying cause of death field: *ICD-10* codes for unintentional fire–related deaths: X00-X19	Only the deaths of people residing in Barcelona are available. People who died in Barcelona are selected regardless of where the episode of injury took place, since this information is not available. The RMB does not have information on injuries, so it is not possible to define mortality indicators based on diagnostic codes, such as TBI fatalities.
43_Fatal_Unintent-MVT	E	Deaths with any of the following codes in the underlying cause of death field: *ICD-10* codes for unintentional motor vehicle traffic-related deaths: [V02-V04] (.1,.9), V09.2, [V12-V14] (.3-.9), V19 (.4-.6), [V20-V28] (.3-.9), [V29-V79] (.4-.9), V80 (.3-.5), V81.1, V82.1, [V83-V86] (.0-.3), V87 (.0-.8), V89.2	Only the deaths of people residing in Barcelona are available. People who died in Barcelona are selected regardless of where the episode of injury took place, since this information is not available. The RMB does not have information on injuries, so it is not possible to define mortality indicators based on diagnostic codes, such as TBI fatalities.
44_Fatal_Unintent-Nondrug	E	Deaths with any of the following codes in the underlying cause of death field: *ICD-10* codes for unintentional nondrug-related deaths: X45-X49	Only the deaths of people residing in Barcelona are available. People who died in Barcelona are selected regardless of where the episode of injury took place, since this information is not available. The RMB does not have information on injuries, so it is not possible to define mortality indicators based on diagnostic codes, such as TBI fatalities.
45_Fatal_Unintent-Drug	E	Deaths with any of the following codes in the underlying cause of death field: *ICD-10* codes for unintentional drug-related deaths: X40-X44	Only the deaths of people residing in Barcelona are available. People who died in Barcelona are selected regardless of where the episode of injury took place, since this information is not available. The RMB does not have information on injuries, so it is not possible to define mortality indicators based on diagnostic codes, such as TBI fatalities.
46_Fatal_Unintent-Suffocation	E	Deaths with any of the following codes in the underlying cause of death field: *ICD-10* codes for unintentional suffocation-related deaths: W75-W84	Only the deaths of people residing in Barcelona are available. People who died in Barcelona are selected regardless of where the episode of injury took place, since this information is not available. The RMB does not have information on injuries, so it is not possible to define mortality indicators based on diagnostic codes, such as TBI fatalities.
47_Suicide	E	Deaths with any of the following codes in the underlying cause of death field: *ICD-10* codes for suicide: X60-X84, Y87.0, U03	Only the deaths of people residing in Barcelona are available. People who died in Barcelona are selected regardless of where the episode of injury took place, since this information is not available. The RMB does not have information on injuries, so it is not possible to define mortality indicators based on diagnostic codes, such as TBI fatalities.
48_Homicide	E	Deaths with any of the following codes in the underlying cause of death field: *ICD-10* codes for homicide: X85-Y09, Y87.1, U01-U02	Only the deaths of people residing in Barcelona are available. People who died in Barcelona are selected regardless of where the episode of injury took place, since this information is not available. The RMB does not have information on injuries, so it is not possible to define mortality indicators based on diagnostic codes, such as TBI fatalities.
49_Fatal_RTI	F^o^	All deaths	The IMLCFC^p^ selects the cases and includes some cases in which the CBD^q^ code according to the judicial mortality register of Barcelona is a code that does not correspond to traffic, such as V43.0, V29.3, and V79.3.
50_Fatal_Pedestrian	F	Deaths selected according to information about the type of user from the 2 sources from which it is fed: GUB and/or IMLCFC; based on the information on the vehicle in which the person was traveling at the time of the collision or by which they were hit and the position they occupied in relation to the vehicle, the type of user variable is generated using the following categories: pedestrian, passenger car user, motorcyclist, cyclist, van or truck user, bus user, and other user	The IMLCFC selects the cases and includes some cases in which the CBD code according to the judicial mortality register of Barcelona is a code that does not correspond to traffic, such as V43.0, V29.3, and V79.3.
51_Fatal_Cyclist	F	Deaths selected according to information about the type of user from the 2 sources from which it is fed: GUB and/or IMLCFC; based on the information on the vehicle in which the person was traveling at the time of the collision or by which they were hit and the position they occupied in relation to the vehicle, the type of user variable is generated using the following categories: pedestrian, passenger car user, motorcyclist, cyclist, van or truck user, bus user, and other user	The IMLCFC selects the cases and includes some cases in which the CBD code according to the judicial mortality register of Barcelona is a code that does not correspond to traffic, such as V43.0, V29.3, and V79.3.
52_Fatal_Motorcyclist	F	Deaths selected according to information about the type of user from the 2 sources from which it is fed: GUB and/or IMLCFC; based on the information on the vehicle in which the person was traveling at the time of the collision or by which they were hit and the position they occupied in relation to the vehicle, the type of user variable is generated using the following categories: pedestrian, passenger car user, motorcyclist, cyclist, van or truck user, bus user, and other user	The IMLCFC selects the cases and includes some cases in which the CBD code according to the judicial mortality register of Barcelona is a code that does not correspond to traffic, such as V43.0, V29.3, and V79.3.
53_Fatal_TBI-RTI	F	Deaths with any of the following codes in any diagnosis field: *ICD-10-CM* diagnosis codes proposed for TBIs: S02.0, S02.1; S02.80, S02.81, S02.82, S02.91; S04.02, S04.03, S04.04; S06; S07.1; T74.4	The IMLCFC selects the cases and includes some cases in which the CBD code according to the judicial mortality register of Barcelona is a code that does not correspond to traffic, such as V43.0, V29.3, and V79.3.
54_RTI_Colisions	G^r^	For people who were injured in the same collision, information about the collision summarized in a single record	N/A^s^
55_RTI_Injured_People	H^t^	All injured people	N/A
56_RTI_Injured_Pedestrian	H	People selected according to information about the type of user from the GUB; based on the information on the vehicle in which the person was traveling at the time of the collision or by which they were hit and the position they occupied in relation to the vehicle, the type of user variable is generated using the following categories: pedestrian, passenger car user, motorcyclist, cyclist, van or truck user, bus user, and other user	N/A
57_RTI_Injured_PedestrianTransport	H	People selected according to information about the type of user from the GUB; based on the information on the vehicle in which the person was traveling at the time of the collision or by which they were hit and the position they occupied in relation to the vehicle, the type of user variable is generated using the following categories: pedestrian, passenger car user, motorcyclist, cyclist, van or truck user, bus user, and other user	N/A
58_RTI_Injured_Motorcyclist	H	People selected according to information about the type of user from the GUB; based on the information on the vehicle in which the person was traveling at the time of the collision or by which they were hit and the position they occupied in relation to the vehicle, the type of user variable is generated using the following categories: pedestrian, passenger car user, motorcyclist, cyclist, van or truck user, bus user, and other user	N/A
59_RTI_Injured_Cyclists	H	People selected according to information about the type of user from the GUB; based on the information on the vehicle in which the person was traveling at the time of the collision or by which they were hit and the position they occupied in relation to the vehicle, the type of user variable is generated using the following categories: pedestrian, passenger car user, motorcyclist, cyclist, van or truck user, bus user, and other user	N/A
60_RTI_Injured_Car	H	People selected according to information about the type of user from the GUB; based on the information on the vehicle in which the person was traveling at the time of the collision or by which they were hit and the position they occupied in relation to the vehicle, the type of user variable is generated using the following categories: pedestrian, passenger car user, motorcyclist, cyclist, van or truck user, bus user, and other user	N/A

a Only codes (both diagnostic and external cause) ending in A, B, C, or missing (reflects initial encounter, active treatment) were included.

b Episodes of nonfatal injuries treated in the hospital emergency departments of Barcelona, for both residents and nonresidents of the city.

c CMBD-AH: Register of the Minimum Basic Data Set of hospital discharges.

d Episodes of nonfatal injuries discharged after admission to a hospital in Barcelona for both residents and nonresidents of the city.

e MAIS: Maximum Abbreviated Injury Score.

f *ICD-10-CM: International Classification of Diseases, Tenth Revision, Clinical Modification.*

g MVT: motor vehicle traffic.

h TBI: traumatic brain injury.

i CDC: Centers for Disease Control and Prevention.

j RTI: road traffic injury.

k Episodes of nonfatal injuries due to traffic collisions treated in the hospital emergency departments of Barcelona, for both residents and nonresidentsof the city.

l Episodes of nonfatal injuries due to traffic collisions discharged after admission to a hospital in Barcelona, for both residents and nonresidents of the city.

m Individuals residing in Barcelona who died from injuries due to external causes within the city.

n RMB: Barcelona Mortality Register.

o Individuals, both residents and nonresidents of Barcelona, who died in Barcelona due to traffic collision–related injuries.

p IMLCFC: L'Institut de Medicina Legal i Ciències Forenses de Catalunya.

q CBD: basic cause of death.

r Traffic collisions in Barcelona where a person, either a resident or nonresident of the city, has been injured (requiring some form of medicalassistance).

s Not applicable.

t Individuals, both residents and nonresidents of Barcelona, injured in traffic collisions in Barcelona (requiring some form of medical assistance).

Table 4 provides a comprehensive overview of the 60 indicators used by the BISS, detailing their corresponding data sources, databases, and injury subsets from which they are derived. It also specifies the inclusion and definition criteria applied to each database, ensuring clarity and consistency in categorization and monitoring. Complementing this, Table 4 outlines the specific definition criteria for each indicator, offering a detailed account of how they are constructed and classified, including any known limitations. This table serves as key references for understanding the methodological framework and scope of the BISS injury surveillance system.

### Quantitative Variables

Several continuous or quantitative variables in the dataset were categorized or summarized for analysis.

Age was analyzed in age groups rather than as a continuous variable. We used the following standard age bands: 0‐14 years, 15‐24 years, 25‐44 years, 45‐64 years, 65‐74 years, and ≥75 years. These groupings align with public health reporting standards and facilitate age-specific rate calculations and comparisons across demographic subpopulations.

Injury severity (as derived from *ICD-10-CM* diagnoses using Abbreviated Injury Score [AIS] or MAIS scores) was dichotomized into severe/critical versus nonsevere. Specifically, injuries with MAIS≥3 were considered “severe” (including critical injuries), while those with MAIS<3 were considered moderate (or minor) injuries. This threshold is commonly used in injury epidemiology to identify serious injuries and was chosen based on international definitions. Some indicators (eg, indicators 2, 12, 24, 32) specifically track the subset of cases meeting this MAIS≥3 criterion.

Time variables such as dates (admission date, discharge date, and collision date) were mainly used for record criteria and calculating durations (eg, length of hospital stay, time trends by year). For annual surveillance reporting, we aggregated data by calendar year. No seasonal nor monthly analyses are presented in this paper, so dates were not explicitly categorized by month or season in this analysis.

Population denominators for rate calculations (number of residents of Barcelona) were treated as continuous counts but were effectively fixed values for each year and each demographic stratum. For standardization, we used the average population of Barcelona across the analysis period as a standard population.

All other variables (eg, sex , mechanism categories, intent) were analyzed as categorical variables, with categories as defined in Table 3. For instance, mechanism of injury has categories like traffic, falls, and burns; intentionality has categories such as unintentional, assault, and self-harm. These categorizations were predetermined based on public health definitions and the coding structure. Grouping of continuous data (eg, age, injury severity scores) was done to enhance interpretability and align with existing frameworks for injury surveillance.

### Bias

Because this study was based on routinely collected data, we considered several potential sources of bias and took steps to address them where possible.

#### Selection Bias

The BISS was designed to be population-based and exhaustive for the defined inclusion criteria, minimizing selection bias. All eligible injury events recorded in the source databases were included (no sampling). However, by design, we excluded minor injuries not captured in hospital or police records, which could bias the overall injury counts toward more severe cases. We acknowledge that the current system underrepresents minor injuries (those only seen in primary care or not treated), an issue we plan to address in future expansions of BISS. In terms of geographic inclusion, hospital records included injuries treated in Barcelona hospitals even if the injury occurred outside the city; thus, there was potential overinclusion of cases not actually stemming from Barcelona incidents. To mitigate this, for mortality, we restricted to city residents, and for nonfatal indicators, we interpreted results with caution, noting that some hospital-treated injuries might be from outside incidents (this is listed as a limitation in relevant indicators, and population rate denominators use city residents who could slightly overestimate true incidence). Conversely, some injuries that occurred in Barcelona may have been treated outside the city (eg, in neighboring municipalities), leading to underascertainment of certain cases. This was likely minimal for serious injuries given the concentration of tertiary hospitals in Barcelona.

#### Information Bias (Misclassification)

We relied on coding in administrative data, which could have led to misclassification of injuries or causes (eg, errors in *ICD-10* coding). To address this, we used previously validated coding algorithms and standard definitions for identifying injury mechanisms and outcomes, and we referenced validation studies of *ICD* coding in Spain when available. For example, the definition of traffic injury cases was based on a published algorithm [26], and all cause-of-death coding followed official mortality coding standards, reducing misclassification of injury deaths. Still, coding inconsistencies were possible (eg, variation in external cause coding practices between hospitals). The inclusion of multiple sources and the focus on broad categories of injuries (rather than single *ICD* codes) helped dilute some random misclassification. We did not have access to independent verification of each case’s details, so some misclassification bias (in injury mechanism or severity) may remain.

#### Confounding

Since this was primarily a descriptive study, confounding (as in risk factor/outcome relationships) was not a major concern. We were not examining an exposure-outcome causal hypothesis but rather summarizing injury occurrence. Therefore, we did not need to control for confounders via stratification or modeling. However, when comparing subgroups (eg, injury rates by sex), we used age-standardized rates to account for confounding by age structure in sex comparisons.

#### Summary of Bias

In summary, the design inherently covered the population of interest and used robust data sources, which helped mitigate selection bias. We made efforts to ensure consistency and accuracy in case definitions to minimize information bias.

### Study Size

No a priori sample size calculation was needed because this study included the entire target population of injury events meeting the inclusion criteria in 2018 through 2024. The effective sample size was determined by the number of eligible injury cases recorded in the data sources. By leveraging whole-population data, the study aimed for comprehensive coverage rather than a targeted enrollment number. The study size was thus fixed by data availability: All relevant records for the year were included. This yielded sufficient numbers to produce stable estimate rates for most indicators at the city level. Where certain subcategory counts were small (for rare causes of injury or specific subpopulations), the results were still reported but interpreted with caution.

### Statistical Methods

All statistical methods applied in this study were descriptive and were planned with the goal of comprehensive surveillance reporting. We did not use inferential statistical tests or complex models; instead, we produced frequency distributions and rate calculations as described in the following paragraphs.

We generated annual summary tables of injury counts and proportions stratified by key variables. For example, we tabulated the number of injury cases by sex, age group, injury mechanism, and injury severity. In these tables, each cell shows the absolute number of cases and its percentage of the column or row total (eg, the proportion of all injuries that falls represent or the percentage of injuries occurring in a given age group). Separate tabulations were done for males and females to facilitate sex-specific insights.

For each of the defined indicators, we calculated the annual incidence rate per 100,000 population. The population denominators were drawn from the Barcelona Municipal Register of Inhabitants (Padron) for the corresponding year. We produced two main types of rate tables for each indicator: (1) overall and sex-specific rates (including crude and age-standardized rates for males and females) and (2) age-specific rates by sex. Age standardization was done using the direct method and the combined 2018‐2024 Barcelona population as the standard, to adjust for differences in age distribution when comparing sexes.

There were very few missing data for core variables due to the nature of the sources (eg, age and sex are almost always recorded in administrative data). If any key variable was missing, those records were still included in overall counts but excluded from stratified analyses requiring that variable. We did not need to apply any imputation, as missingness was minimal and not central to the indicators.

We did not perform analyses of subgroups or interactions beyond the stratifications by sex and age already described. The design of the indicators inherently stratified certain subgroups. We did not explicitly test interactions or perform subgroup hypothesis tests, since our focus was on reporting descriptive metrics rather than estimating effect sizes.

Given that our study was descriptive, we did not conduct formal sensitivity analyses. However, we did examine whether results were consistent over the time series (2018‐2024) as an informal check.

All analyses were performed using Stata 16 statistical software. Data cleaning and rate calculations were done using standard Stata procedures and validated by cross-checking selected results with an independent calculation. The statistical outputs (tables and figures) were then compiled for reporting, with no adjustments for multiple comparisons and no derivation of *P* values.

### Ethical Considerations

This study was based exclusively on anonymized secondary data collected within the routine mandate of public health authorities, which did not require review by a research ethics committee. All data were processed in compliance with applicable data protection regulations.

### Data Access and Cleaning Methods

Access to the underlying data was governed by institutional agreements, but within the research team, the investigators had full access to all relevant de-identified records used to create the study datasets. Specifically, the Public Health Agency of Barcelona has authorized access to the CMBD and mortality databases for surveillance purposes as well as to the police collision database through a collaboration with the city police. All data were handled in compliance with privacy regulations. Therefore, although raw data cannot be made public, the authors (analysts) could use the complete datasets for the purposes of this study. We also note that details on how to access supplemental materials (eg, coding lists or aggregate data tables) are provided in the manuscript or Multimedia Appendix 1 as needed.

Data cleaning procedures were carried out to ensure a high-quality analytic dataset. Key steps included filtering and case selection, merging and deduplication, coding harmonization, missing or inconsistent values, and linkage quality (if applicable).

For filtering and case selection, we applied the inclusion criteria algorithms (per Table 1) to each source to extract injury cases. This involved querying diagnosis and external cause code fields for relevant *ICD-10-CM* codes, selecting only Barcelona residents for the mortality dataset, and selecting only traffic-related cases for the BISS-RTI subsets using cause codes. We verified that these filters were correctly implemented by cross-checking data.

For merging and deduplication, records are uniquely identified within each data source. In the health datasets (ED and HOSP), it is possible that the same patient’s care could generate multiple records (eg, an ED visit followed by an admission). Currently, due to the unavailability of a common patient ID, it was not possible to avoid double counting. We treated ED and HOSP data as separate strata and did not merge them at the patient level; an ED visit that became an admission would appear in both ED and HOSP databases. It was expected that, in the future, patient ID will be available for BISS and consequently, no double-counting would occur across ED versus HOSP indicators, making them mutually exclusive. In contrast, we ensured that deaths were only counted in the mortality datasets and not simultaneously in ED or HOSP (for example, if a person died in hospital, that case is recorded in the mortality register and in the HOSP data; for BISS reporting, we counted it as a death, not a nonfatal hospitalization). In the police data, each injured person in a collision is a separate record; if one person had multiple injury-causing collisions, they would appear multiple times, but each corresponds to a distinct event (this is consistent with the definition of counting episodes, not unique individuals).

Regarding coding harmonization, we standardized variable coding across data sources. For instance, we created common categorical variables for mechanism and intent of injury using *ICD* codes from health data and analogous categories from police data. We also mapped the police-defined road user categories to match the categories used in health data where possible. All *ICD-10* and *ICD-10-CM* codes were checked against their descriptions to confirm they were correctly grouped under each mechanism or intent category.

We also performed checks for impossible, inconsistent, or missing values (eg, negative ages, admission dates after discharge dates). No systematic issues were found; any obvious data errors were minimal, and those records were either corrected if possible or excluded if not critical. For example, if an ED record was missing age or sex, it would not contribute to age- or sex-specific analyses but would still be counted in total injuries.

Regarding linkage quality, in the RMB-CT (traffic fatality), data linkage between sources (forensic, police, and death certificate) is managed by a deterministic match (using identifiers like national ID, name, date of birth, and date of incident). The quality of these linkages is maintained by the Public Health Agency’s longstanding procedures; for this study, we did not perform independent linkage, but we trusted the quality control in the existing RMB-CT process. All traffic deaths included have been vetted through that system. We did not link the police nonfatal records with hospital records due to lack of identifiers; thus, no probabilistic linkage procedure was required beyond what is done within the RMB-CT for fatalities.

Throughout, the data cleaning process was thoroughly documented. The code used for data processing and analysis is available upon request, and the study protocol and variable code lists are provided in Multimedia Appendix 1. These steps ensured that the final analytical datasets were accurate representations of the source data, with a harmonized structure and ready for calculating the injury indicators.

### Stakeholders Involved in the Injury Surveillance System

A wide range of stakeholders were involved in the development, implementation, and use of the injury surveillance system. These included public health officials, hospital and emergency care providers, epidemiologists, injury prevention specialists, data analysts, policy-makers, researchers, and representatives from affected communities. Each stakeholder group played a critical role in ensuring that the system generates accurate, relevant, and timely data to inform prevention strategies and health policies. Their active engagement enhanced the system’s relevance, facilitated data interpretation, and support the translation of findings into effective public health action.

### Evaluation of the Surveillance System

The surveillance system will be evaluated following CDC guidelines to assess its performance and alignment with public health objectives. Key attributes—such as usefulness, simplicity, flexibility, data quality, acceptability, sensitivity, predictive value, representativeness, timeliness, and stability—will be analyzed to identify strengths and areas for improvement. Evaluation findings will guide targeted recommendations, developed in consensus with stakeholders, and communicated in formats adapted to relevant audiences to support system enhancement.

### Dissemination of Surveillance System Results

The dissemination of results from the BISS is a critical step to ensure that collected data translate into effective public health action. Results will be shared systematically with key stakeholders. The planned dissemination includes a web-based dashboard featuring indicators displayed in tables, graphs, and maps across multiple years; reports, infographics, and policy briefs; peer-reviewed publications; and oral and poster presentations, including those at individual, community, and professional meetings. Efforts will be made to ensure that the results are communicated in a timely, accessible, and actionable manner, facilitating data-driven decision-making and strengthening injury prevention strategies across sectors.

## Results

### Age and Sex Distribution

Overall, 123,420 nonfatal injury episodes were recorded in 2024 in hospital EDs, of which 99,379 (80.5%) occurred among Barcelona residents. ED episodes were slightly more frequent in females than in males, both in total episodes (63,108 vs 60,312) and among residents (52,323 vs 47,056). A total of 18,749 nonfatal hospitalized injury episodes were recorded in 2024, of which 14,319 (76.4%) were among Barcelona residents. Hospitalized episodes were also somewhat more frequent in females than in males, both in total episodes (9645 vs 9104) and among residents (7916 vs 6403). In contrast, mortality data (year 2023) included 695 fatal injuries, all among Barcelona residents, of whom 388 were males and 307 were females (Table 5).

**Table 5.** Number, age-specific and age-adjusted rates by sex for nonfatal emergency department (ED) and hospitalized injury episodes as well as injury-related mortality from the Barcelona Injury Surveillance System (BISS).

Age group (years) by sex^a^	Nonfatal ED episodes (injury subset A: ED^b^ database) in 2024			Nonfatal hospitalized episodes (injury subset C: HOSP^c^ database) in 2024			Fatally injured people (injury subset E: MORTALITY^d^ database) in 2023	
	Total injuries, n	Residents in Barcelona		Total injuries, n	Residents in Barcelona		Residents in Barcelona	
		Injuries, n	Age-specific rate (per 100,000)^e^		Injuries, n	Age-specific rate (per 100,000)^e^	Injuries, n	Age-specific rate (per 100,000)^e^
Male								
0‐14	6874	5868	5929.1	540	246	249	1	1
15‐24	8258	5325	6072.3	653	332	379	13	15.2
25‐44	19,711	14,305	5368.1	1754	1048	393	43	16.4
45‐64	12,650	9955	4498.2	2059	1270	574	78	35.5
65‐74	4281	3641	5074.3	979	735	1024	36	50.6
≥75	8468	7897	10,983.5	3119	2772	3855	217	306.6
Unknown	70	65	N/A^f^	0	0	N/A	0	N/A
Total	60,312	47,056	5743.9 (AR^g^=5873.1)	9104	6403	782.7 (AR=875.0)	388	47.9 (AR=55.6)
Female								
0‐14	4806	4067	4356.7	356	176	189	1	1.1
15‐24	6359	4014	4774.3	398	260	309	4	4.9
25‐44	13,869	10,449	3957.3	775	514	195	11	4.2
45‐64	13,197	10,768	4522.8	1320	908	381	28	11.8
65‐74	7080	6145	6595.5	1058	838	899	17	18.4
≥75	17,787	16,873	13,953.0	5738	5220	4317	246	205.6
Unknown	10	7	N/A	0	0	N/A	0	N/A
Total	63,108	52,323	5854.2 (AR=5619.6)	9645	7916	885.8 (AR=788.5)	307	34.6 (AR=30.0)

a For residents of Barcelona, the sex of 14 injured people was unknown, and the ages of 7 females and 65 males were also unknown. For nonresidents of Barcelona, the sex of 5 injured people was unknown, and the ages of 3 femals and 5 males were also unknown.

b Emergency department visit data.

c Hospital discharge data.

d Injury-related mortality data.

e Rates were calculated using as the numerator only men and women for whom age information was available.

f N/A: not applicable.

g AR: age-adjusted rate.

By sex, crude resident rates for nonfatal injuries were slightly higher for females than for males for both ED episodes (5854.2 vs 5743.9 per 100,000) and hospitalized episodes (885.8 vs 782.7 per 100,000). However, age-adjusted rates were higher for ED episodes in males younger than 45 years and hospitalized episodes in males younger than 75 years. For mortality, both crude and age-adjusted rates were clearly higher in males than in females (47.9 vs 34.6 per 100,000 and 55.6 vs 30.0 per 100,000, respectively), indicating a greater fatal injury burden among males.

Injury rates varied markedly by age. ED rates were higher in males at younger ages but higher in females at older ages, especially from the age of 65 years onward. Hospitalized injuries showed a similar pattern, with the highest rates in the oldest age groups, particularly among females. Fatal injury rates increased sharply with age in both sexes and were highest among adults aged 75 years or older.

### Injury Mechanism

Table 6 shows the distribution of injury episodes and deaths by injury mechanism in the ED, hospitalized, and mortality data sources. Missing or unspecified information was substantial, particularly in the ED database, where episodes without an external cause code accounted for 63.6% (38,337/60,312) of records for males and 62.8% (39,662/63,108) for females; the corresponding proportions in hospitalized episodes were 20.4% (1854/9104) and 16.5% (1591/9645), respectively, while nonspecific mechanisms also represented a relevant share across all sources, especially mortality records. Among specified mechanisms, unintentional falls were the most frequent in both ED and hospitalized data and were consistently more common in females than in males (ED: 7052/63,108, 11.2% vs 4761/60,312, 7.9%; hospitalized: 4583/9645, 47.5% vs 2890/9104, 31.7%). Intentional self-harm was also frequent and occurred at higher proportions for females in the nonfatal data (ED: 2260/63,108, 3.6% vs 1515/60,312, 2.5%; hospitalized: 508/9645, 5.3% vs 311/9104, 3.4%), whereas males more often presented with aggression, motor vehicle traffic injuries, and several other unintentional mechanisms. In the mortality data, unintentional asphyxia was the leading specified mechanism and was more frequent for females (113/307, 36.8% vs 114/388, 29.4%), while intentional self-harm had the largest sex difference in the opposite direction, with markedly higher proportions among males than females (75/388, 19.3% vs 31/307, 10.1%).

**Table 6.** Number and percentage of nonfatal episodes treated in hospital emergency services, nonfatal episodes that were discharged after hospitalization, and fatally injured people by principal mechanism and sex from the Barcelona Injury Surveillance System (BISS).

Principal mechanism	Nonfatal ED^a^ episodes (injury subset A: ED^b^ database) in 2024				Nonfatal hospitalized episodes (injury subset C: HOSP^c^ database) in 2024				Fatally injured people (injury subset E: MORTALITY^d^ database) in 2023			
	Males (n=60,312), n (%)		Females (n=63,108), n (%)		Males (n=9104), n (%)		Females (n=9645), n (%)		Males (n=388), n (%)		Females (n=307), n (%)	
Intentional self-harm	1515 (2.5)		2260 (3.6)		311 (3.4)		508 (5.3)		75 (19.3)		31 (10.1)	
Aggression	882 (1.5)		381 (0.6)		289 (3.2)		58 (0.6)		9 (2.3)		2 (0.7)	
Unintentional fall	4761 (7.9)		7052 (11.2)		2890 (31.7)		4583 (47.50)		39 (10.1)		35 (11.4)	
Traffic with a motor vehicle, unintentional	311 (0.5)		287 (0.5)		251 (2.8)		155 (1.6)		17 (4.4)		3 (1)	
Cyclist nontraffic/nonmotor vehicle, unintentional	265 (0.4)		102 (0.2)		121 (1.3)		34 (0.4)		—^e^		—	
Pedestrian not traffic/no vehicle motor, unintentional	13 (0)		33 (0.1)		19 (0.2)		30 (0.3)		2 (0.5)		0 (0)	
Nontraffic with a motor vehicle, unintentional	15 (0)		14 (0)		12 (0.1)		7 (0.1)		1 (0.3)		0 (0)	
Other transport, unintentional	20 (0)		22 (0)		18 (0.2)		12 (0.1)		—		—	
Exposure to fire/smoke, unintentional	215 (0.4)		270 (0.4)		349 (3.8)		278 (2.9)		1 (0.3)		5 (1.6)	
Unintentional cutting or piercing	518 (0.9)		304 (0.5)		107 (1.2)		23 (0.2)		0 (0)		1 (0.3)	
Unintentional overexertion	633 (1)		735 (1.2)		91 (1)		102 (1.1)		—		—	
Hit by or against object, unintentional	801 (1.3)		611 (1)		155 (1.7)		73 (0.8)		—		—	
Drowning/submersion, unintentional	0 (0)		0 (0)		4 (0)		0 (0)		4 (1)		3 (1)	
Machinery, unintentional	51 (0.1)		9 (0)		105 (1.2)		9 (0.1)		—		—	
Natural/environmental, unintentional	217 (0.4)		312 (0.5)		55 (0.6)		43 (0.4)		4 (1)		3 (1)	
Asphyxia, unintentional	178 (0.3)		171 (0.3)		172 (1.9)		102 (1.1)		114 (29.4)		113 (36.8)	
Drug poisoning, unintentional	754 (1.3)		485 (0.8)		241 (2.6)		235 (2.4)		23 (5.9)		8 (2.6)	
Nondrug poisoning, unintentional	1640 (2.7)		1190 (1.9)		90 (1)		61 (0.6)		5 (1.3)		2 (0.7)	
Other specified, unintentional	3400 (5.6)		2981 (4.7)		1259 (13.8)		946 (9.8)		0 (0)		2 (0.7)	
Nonspecific	5786 (9.6)		6227 (9.9)		711 (7.8)		795 (8.3)		94 (24.2)		99 (32.2)	
No external cause code	38,337 (63.6)		39,662 (62.8)		1854 (20.4)		1591 (16.5)		—		—	

a ED: emergency department.

b Emergency department visit data.

c Hospital discharge data.

d Injury-related mortality data.

e Data not available in the mortality register.

### Nature of Injury, Anatomical Region, and Injury Severity

Table 7 shows the distribution of nonfatal injury episodes in 2024 by type of injury, anatomical region, and injury severity, separately for ED and hospitalized episodes and for males and females. In both care settings, substantial proportions of cases were coded as unspecified injury, particularly in ED episodes, where this category accounted for 37.3% (22,417/60,312) of episodes in males and 40.4% (25,470/63,108) of episodes in females, compared with 4% (362/9104) and 4.3% (411/9645), respectively, among hospitalized episodes. Among specified injury types, fractures were more frequent in females than in males and were especially prominent among hospitalized episodes (4855/9645, 50.3% in females vs 3812/9104, 41.9% in males), whereas open wounds were more common in men in both the ED and hospitalized data. Extremities and head and neck were the most frequently affected anatomical regions in both sexes, although trunk injuries represented a larger proportion of hospitalized episodes in males than in females. Regarding injury severity, most episodes were classified as nonsevere, but severe injuries accounted for a considerable proportion of hospitalized episodes and were slightly more frequent in females than in males (4540/9645, 47.1% vs 4025/9104, 44.2%), while the proportion of episodes with unknown severity remained notable in both data sources.

**Table 7.** Nature of injury, anatomical region, and injury severity for episodes treated in hospital emergency services and discharged after hospitalization, by sex in the Barcelona Injury Surveillance System (BISS).

Variables	Nonfatal ED^a^ episodes (injury subset A: ED^b^ database) in 2024^c^		Nonfatal hospitalized episodes (injury subset C: HOSP^d^ database) in 2024^c^	
	Males (n=60,312), n (%)	Females (n=63,108), n (%)	Males (n=9104), n (%)	Females (n=9645), n (%)
Type of injury				
Burns and corrosions	1093 (1.8)	1061 (1.7)	425 (4.7)	321 (3.3)
Dislocation	1763 (2.9)	1300 (2.1)	217 (2.4)	117 (1.2)
Effect of foreign body entering orifice	3529 (5.9)	3100 (4.9)	1468 (16.1)	1111 (11.5)
Fracture	9071 (15)	11,081 (17.6)	3812 (41.9)	4855 (50.3)
Internal organ injury	5594 (9.3)	6399 (10.1)	2111 (23.2)	1927 (20)
Open wound	7520 (12.5)	4641 (7.4)	1093 (12)	679 (7)
Other effects of external causes	2046 (3.4)	1872 (3)	49 (0.5)	55 (0.6)
Poisoning	1617 (2.7)	1508 (2.4)	542 (6)	731 (7.6)
Superficial and contusion	9097 (15.1)	9147 (14.5)	861 (9.5)	1012 (10.5)
Toxic effects	2238 (3.7)	1843 (2.9)	233 (2.6)	147 (1.5)
Other specified injury^e^	14,122 (23.3)	15,127 (23.9)	607 (6.7)	298 (3.1)
Unspecified injury	22,471 (37.3)	25,470 (40.4)	362 (4)	411 (4.3)
Anatomical regions				
Extremities	25,630 (42.5)	26,494 (42)	3997 (43.9)	4831 (50.1)
Head and neck	20,832 (34.5)	22,467 (35.6)	2625 (28.8)	2471 (25.6)
Spine and back	725 (1.2)	1012 (1.6)	475 (5.2)	488 (5.1)
Trunk	4713 (7.8)	5342 (8.5)	2800 (30.8)	2297 (23.8)
Unclassified region	5511 (9.1)	4840 (7.7)	923 (10.1)	977 (10.1)
Nonspecific region	12,267 (20.3)	11,736 (18.6)	551 (6.1)	459 (4.8)
Injury severity				
Severe (MAIS^f^≥3)	19,915 (33)	19,206 (30.4)	4025 (44.2)	4540 (47.1)
Not severe (MAIS<3)	33,490 (55.5)	37,294 (59.1)	3059 (33.6)	3195 (33.1)
Unknown^g^	6907 (11.5)	6608 (10.5)	2020 (22.2)	1910 (19.8)

a ED: emergency department.

b Emergency department visit data.

c Each category of type of injury and anatomical region shows the number of people with an injury of that type or in that anatomical region; therefore, percentages sum to more than 100%, as one person may have multiple injuries affecting multiple regions.

d Hospital discharge data.

e Amputation, blood vessel, crushing, sprains and strains, injury to nerves, injury to muscles and tendons, other injury.

f MAIS: Maximum Abbreviated Injury Score.

g Includes episodes with diagnosis codes considered traumatic injuries according to the study definition and Centers for Disease Control and Prevention criteria but not mapped by the ICD Programs for Injury Categorization in R (ICDPIC-R) [27] because they do not correspond to anatomical injuries.

### Indicators

Table 8 shows a selection of indicators included in the BISS. Age-adjusted rates of all injuries, severe injuries, and traumatic brain injury were higher in males than in females in both the ED and hospitalization data. In the ED data, males had higher rates of all injuries (5873.1 vs 5619.6), severe injuries (1896.6 vs 1691.3), and traumatic brain injury (1120.8 vs 917.0). However, mechanism-specific ED indicators (indicators 3‐9) should be interpreted with caution because of the high proportion of missing values. In hospitalized episodes, males also had higher age-adjusted rates of all injuries (875.0 vs 788.5), severe injuries (283.0 vs 258.6), and traumatic brain injury (230.9 vs 187.3), whereas females showed higher rates of fall-related injuries (401.8 vs 341.4), long-bone fractures (253.5 vs 181.0), fall-related hip fractures among people aged ≥65 years (115.6 vs 69.5), and self-harm (50.4 vs 30.5). In contrast, assault-related hospitalization rates were higher in males (19.7 vs 4.8). Mortality rates were consistently higher in males, both overall (55.6 vs 30.0) and for specific causes, particularly unintentional suffocation (17.9 vs 10.8), self-harm/suicide (9.4 vs 3.3), falls (5.7 vs 3.4), drug poisoning/overdose (2.8 vs 0.9), and traffic-related injuries (2.1 vs 0.3).

**Table 8.** Barcelona Injury Surveillance System (BISS) indicator results by sex for people residing in Barcelona.

Indicator	Nonfatal ED^a^ episodes (injury subset A: ED^b^ database) in 2024						Nonfatal hospitalized episodes (injury subset C: HOSP^c^ database) in 2024						Fatally injured people (injury subset E: MORTALITY^d^ database) in 2023					
	Males			Females			Males			Females			Males			Females		
	Injuries, n	Crude rate (per 100,000)	Age-adjusted rate (per 100,000)	Injuries, n	Crude rate (per 100,000)	Age-adjusted rate (per 100,000)	Injuries, n	Crude rate (per 100,000)	Age-adjusted rate (per 100,000)	Injuries, n	Crude rate (per 100,000)	Age-adjusted rate (per 100,000)	Injuries, n	Crude rate (per 100,000)	Age-adjusted rate (per 100,000)	Injuries, n	Crude rate (per 100,000)	Age-adjusted rate (per 100,000)
1/11/39: All injuries	46,991	5743.90	5873.10	52,316	5854.20	5619.60	6403	782.7	875	7916	885.8	788.5	388	47.9	55.6	307	34.6	30
2/12: Severe injuries	15,228	1861.40	1896.60	15,867	1775.50	1691.30	2053	250.9	283	2644	295.9	258.6	—^e^	—	—	—	—	—
3/13/48: Assault/homicide	515	63	60	270	30.2	30.8	167	20.4	19.7	43	4.8	4.8	9	1.1	1.1	2	0.2	0.2
4/14/47: Self-harm/suicide	1222	149.4	143.2	1919	214.7	218.8	254	31	30.5	444	49.7	50.4	75	9.3	9.4	31	3.5	3.3
5/15/41: Unintentional fall-related injuries	3911	478.1	521.9	6132	686.2	623.8	2382	291.2	341.4	4149	464.3	401.8	39	4.8	5.7	35	3.9	3.4
6/16/43: Unintentional motor vehicle traffic-related injuries	200	24.4	24	181	20.3	19.8	148	18.1	18.4	95	10.6	10.2	17	2.1	2.1	3	0.3	0.3
7/17/42: Fire-related injuries	113	13.8	13.6	159	17.8	17.8	113	13.8	13.6	105	11.7	11.9	1	0.1	0.1	5	0.6	0.5
8/18/44: Unintentional nondrug poisoning injuries	1230	150.3	146.2	975	109.1	110.1	98	12	12.2	60	6.7	6.6	5	0.6	0.6	2	0.2	0.2
9/19/45: Unintentional drug poisoning injuries/drug overdose	820	100.2	96.5	698	78.1	78.2	188	23	24.2	206	23.1	21.3	23	2.8	2.8	8	0.9	0.9
46: Unintentional suffocation fatalities	—	—	—	—	—	—	—	—	—	—	—	—	114	14.1	17.9	113	12.7	10.8
40: Unintentional drowning fatalities	—	—	—	—	—	—	—	—	—	—	—	—	4	0.5	0.5	3	0.3	0.3
10/20: TBI^f^	8436	1031.2	1120.8	8966	1003.3	917	1624	198.5	230.9	1934	216.4	187.3	—	—	—	—	—	—
21: Long bone fracture	—	—	—	—	—	—	1329	162.4	181	2571	287.7	253.5	—	—	—	—	—	—
22: Fall-related hip fracture in persons ≥65 years old	—	—	—	—	—	—	452	55.2	69.5	1226	137.2	115.6	—	—	—	—	—	—

a ED: emergency department.

b Emergency department visit data.

c Hospital discharge data.

d Injury-related mortality data.

e Data not available in the mortality register.

f TBI: traumatic brain injury.

Self-harm rates were highest among females aged 15 years through 24 years, who showed the greatest burden of all age-sex groups. In contrast, unintentional falls increased steeply with age and were particularly concentrated among older females, with the highest rates observed in those aged ≥65 years, especially among females aged ≥75 years (Table 9).

**Table 9.** Number of nonfatal episodes discharged after hospitalization and age-specific rates by sex for unintentional falls and self-harm related injuries in the resident Barcelona population, as recorded in the Barcelona Injury Surveillance System (BISS) in 2024.

Mechanism by age group (years)	Males		Females	
	Episodes, n	Episodes, age-specific rate × 100,000 population (residents)	Episodes, n	Episodes, age-specific rate × 100,000 population (residents)
Self-harm				
0‐14	3	3	36	38.6
15‐24	53	60.4	130	154.6
25‐44	88	33	102	38.6
45‐64	76	34.3	127	53.3
65‐74	15	20.9	26	27.9
≥75	19	26.4	23	19
Unintentional falls				
0‐14	79	79.8	42	45
15‐24	52	59.3	32	38.1
25‐44	191	71.7	108	40.9
45‐64	354	160	281	118
65‐74	299	416.7	425	456.2
≥75	1407	1956.9	3261	2696.7

### Trends

From 2018 through 2024, nonfatal injury rates showed a dip in 2020 followed by a sustained increase in both sexes. Overall, ED-treated injuries rose slightly for males (+3.4%) and females (+3.2%), whereas severe ED-treated injuries increased markedly (+54.5% and +49.1%, respectively). Discharged injuries also increased in both sexes, with females consistently showing higher rates than males. Fall-related injuries rose sharply and remained substantially higher in females, while self-harm injuries increased in both sexes but increased more among females. In contrast, road traffic injuries declined overall, although rates remained consistently higher in males. Fatal injury rates (2018‐2023) increased in both sexes, especially in males, who also had persistently higher mortality than females throughout the period (Table 10).

**Table 10.** Barcelona Injury Surveillance System (BISS) indicator trends by sex for people residing in Barcelona from 2018 through 2024.

Indicators by sex	2018		2019		2020		2021		2022		2023		2024	
	Episodes, n	Episodes, crude rate (per 100,000)	Episodes, n	Episodes, crude rate (per 100,000)	Episodes, n	Episodes, crude rate (per 100,000)	Episodes, n	Episodes, crude rate (per 100,000)	Episodes, n	Episodes, crude rate (per 100,000)	Episodes, n	Episodes, crude rate (per 100,000)	Episodes, n	Episodes, crude rate (per 100,000)
Males														
Nonfatal ED^a^ injuries														
1_All injuries	43,221	5556.4	45,682	5738.2	32,483	4124.7	38,977	4945.1	42,413	5413.8	45,21	5579.2	46,991	5743.9
2_Severe (MAIS^b^≥3) injuries	9373	1205.0	9974	1252.8	8256	1048.3	11,150	1414.6	13,284	1695.6	14,821	1828.8	15,228	1861.4
Nonfatal discharged injuries														
11_All injuries	4671	600.5	4963	623.4	4536	576	5325	675.6	5761	735.4	5956	734.9	6403	782.7
12_Severe (MAIS≥3) injuries	1372	176.4	1505	189	1458	185.1	1732	219.7	1899	242.4	2118	261.4	2053	250.9
15_Unintentional fall-related injuries	1568	201.6	1442	181.1	1414	179.5	1702	215.9	2160	275.7	2238	276.2	2382	291.2
14_Intentional self-harm injuries	171	22	168	21.1	189	24	174	22.1	218	27.8	190	23.4	254	31
31_Road traffic injuries	472	60.7	484	60.8	352	44.7	462	58.6	404	51.6	377	46.5	450	55
Fatal injuries														
39_All injuries	303	39	296	37.2	366	46.5	336	42.6	355	45.3	388	47.9	N/A^c^	N/A
41_Unintentional fall-related injuries	36	4.6	18	2.3	45	5.7	33	4.2	28	3.6	39	4.8	N/A	N/A
47_Intentional self-harm injuries	64	8.2	59	7.4	76	9.7	59	7.5	69	8.8	75	9.3	N/A	N/A
Females														
Nonfatal ED injuries														
1_All injuries	49,011	5,674.6	51,131	5818.3	36,103	4157.3	41,838	4837.1	46,848	5426.2	49,989	5634.8	52,316	5854.2
2_Severe (MAIS≥3) injuries	10,287	1191.0	10,635	1210.2	8835	1017.4	12,159	1405.8	14,781	1712.0	15,836	1785.0	15,867	1775.5
Nonfatal discharged injuries														
11_All injuries	6330	732.9	6652	756.9	6283	723.5	6866	793.8	6948	804.8	7639	861.1	7916	885.8
12_Severe (MAIS≥3) injuries	1813	209.9	1946	221.4	2031	233.9	2153	248.9	2296	265.9	2717	306.3	2644	295.9
15_Unintentional fall-related injuries	2758	319.3	2678	304.7	2620	301.7	3024	349.6	3528	408.6	4068	458.5	4149	464.3
14_Intentional self-harm injuries	239	27.7	270	30.7	303	34.9	366	42.3	394	45.6	399	45	444	49.7
31_Road traffic injuries	235	27.2	203	23.1	164	18.9	202	23.4	208	24.1	184	20.7	214	23.9
Fatal injuries														
39_All injuries	258	29.9	260	29.6	315	36.3	303	35	318	36.8	307	34.6	N/A	N/A
41_Unintentional fall-related injuries	35	4.1	33	3.8	71	8.2	52	6	34	3.9	35	3.9	N/A	N/A
47_Intentional self-harm injuries	32	3.7	23	2.6	32	3.7	30	3.5	29	3.4	31	3.5	N/A	N/A

a ED: emergency department.

b MAIS: Maximum Abbreviated Injury Score.

c N/A: not available.

### Road Traffic Injuries

Table 11, which includes data from the road traffic injury subsystem and police records (GUB), shows a consistently higher burden of traffic-related injuries among males than among females across most indicators and data sources. All rates were calculated using the resident population as the denominator; therefore, the analysis was restricted to residents. For all injuries combined, males had higher numbers and crude rates than females for nonfatal ED episodes, nonfatal hospitalized episodes, fatalities, and injured people recorded in police data. Severe injuries (MAIS≥3) were also more frequent among males in both ED and hospitalized episodes. Motorcyclist injuries accounted for a substantial share of the burden, particularly among males, with markedly higher counts and rates across all available sources. Cyclist injuries and injuries among users of personal transport devices were also more common in males. In contrast, pedestrian injuries showed a more balanced sex pattern: Although hospitalized pedestrian injuries were more frequent in males, police data recorded more injured female pedestrians than male pedestrians, and pedestrian fatalities were slightly higher among females. Car user injuries had smaller sex differences in the ED and hospitalized data, although police records still indicated higher numbers among males. Finally, traumatic brain injury was more frequent among males than females across ED, hospitalized, and fatal cases. Importantly, these resident-based estimates do not capture the full number of traffic-injured people occurring in the city, as many additional injured individuals were also recorded by the Guàrdia Urbana but were not included in the analytical rates because the denominator was restricted to residents only.

**Table 11.** Barcelona Injury Surveillance System for road traffic injury (BISS-RTI) indicator results by sex for people residing in Barcelona in 2024 RTI fatalities among Barcelona residents due to collisions occurring in Barcelona

Indicator	RTI^a^ nonfatal ED^b^ episodes				RTI nonfatal hospitalized episodes				RTI fatalities^c^				Injured people police data			
	Males		Females		Males		Females		Males		Females		Males		Females	
	n	Crude rate (per 100,000)	n	Crude rate (per 100,000)	n	Crude rate (per 100,000)	n	Crude rate (per 100,000)	n	Crude rate (per 100,000)	n	Crude rate (per 100,000)	n	Crude rate (per 100,000)	n	Crude rate (per 100,000)
23/31/49_All injuries	1839	224.8	1180	132	450	55	214	23.9	5	0.6	3	0.3	3346	409	2354	263.4
24/32_Severe (MAIS^d^≥3) injuries	1155	141.2	668	74.7	221	27	109	12.2	N/A^e^	N/A	N/A	N/A	N/A	N/A	N/A	N/A
25/33/50_Pedestrian injuries	86	10.5	88	9.8	65	7.9	44	4.9	1	0.1	2	0.2	337	41.2	409	45.8
26/34_Pedestrian transport users injuries^f^	121	14.8	66	7.4	41	5	21	2.3	N/A	N/A	N/A	N/A	250	30.6	133	14.9
27/35/51_Cyclist injuries	180	22	67	7.5	42	5.1	12	1.3	1	0.1	1	0.1	335	40.9	149	16.7
28/36/52_Motorcyclist injuries	458	56	181	20.3	222	27.1	55	6.2	2	0.2	0	—^g^	1915	234.1	1006	112.6
29/37_Car user injuries	37	4.5	40	4.5	14	1.7	17	1.9	N/A	N/A	N/A	N/A	425	51.9	314	35.1
30/38/53_TBI^h^	202	24.7	177	19.8	95	11.6	59	6.6	5	0.6	3	0.3	N/A	N/A	N/A	N/A

a RTI: road traffic injury.

b ED: emergency department.

c RTI fatalities among Barcelona residents due to collisions occurring in Barcelona.

d MAIS: Maximum Abbreviated Injury Score.

e N/A: not available.

f Transport examples include skates, skateboards, nonmotorized scooters, and shoes with wheels.

g Not applicable.

h TBI: traumatic brain injury.

## Discussion

This study provides a comprehensive assessment of the burden of nonfatal and fatal injuries in Barcelona using an integrated injury information system based on ED, hospitalization, mortality, and, for road traffic injuries, police data. Several findings deserve emphasis. First, nonfatal injuries were slightly more frequent among females, particularly at older ages, whereas fatal injuries were clearly more frequent among males. Second, falls were the leading specified mechanism in nonfatal care and were concentrated among older females, while self-harm represented a marked burden among young females in hospitalization data but a higher mortality burden among males. Third, from 2018 through 2024, most nonfatal injury indicators increased after the decline observed in 2020, whereas road traffic injuries showed an overall downward trend. Taken together, these findings highlight the heterogeneous distribution of injury burden by sex, age, mechanism, and severity and show the added value of combining multiple routine data sources to identify prevention priorities at the city level.

Our findings also underline the public health relevance of integrated injury surveillance. Surveillance has been defined as the ongoing, systematic collection, analysis, interpretation, and dissemination of health data needed for prevention and control, and WHO injury surveillance guidance similarly emphasizes that the ultimate purpose of injury data collection is to support prevention strategies that reduce death and disability. In this context, this study showed that a multisource system can do more than quantify service use: It can identify high-risk population groups, characterize the severity and mechanisms of injury, and monitor trends over time. This is particularly important in urban settings, where injury risks are shaped by mobility, aging, social context, and health care use and where prevention requires coordinated action across sectors [6,28].

The predominance of falls, especially among older females, is one of the most relevant findings of this study. Falls were the leading specified mechanism in both ED and hospitalized injury data, and rates increased sharply with age, with the highest burden observed in females aged ≥65 years, particularly those aged ≥75 years. This pattern is consistent with previous evidence showing that falls are a major cause of injury-related morbidity and mortality in older adults worldwide. WHO identifies falls as a major public health problem in older populations, and recent studies have shown that frailty substantially increases the long-term risk of fall- and fracture-related hospitalization, particularly among older women. Our findings therefore support the need to frame injury prevention in cities not only around transport safety but also around healthy aging, fall prevention, and the prevention of severe consequences such as fractures, loss of autonomy, and institutionalization [29,30].

Self-harm emerged as another key priority. In our data, hospitalization rates for self-harm were highest among females aged 15 years to 24 years, whereas mortality from self-harm was substantially higher among males. This distinction between nonfatal and fatal burden is important because it suggests that mortality data alone provide an incomplete picture of self-harm epidemiology. The findings are consistent with recent European evidence. In France, hospitalizations for self-harm have increased sharply among adolescent girls and young women since late 2020, and a nationwide registry-based study in Spain reported rising hospitalization rates for nonlethal intentional self-harm between 2018 and 2023, particularly among young women. This pattern is also supported by meta-analytic evidence showing a higher prevalence of self-injurious behaviors among adolescent females. Together, these findings reinforce the need for sex- and age-sensitive prevention strategies that connect injury surveillance with youth mental health services and suicide prevention policies [31,32].

The temporal trends observed in this study are also informative. The decline in nonfatal injuries in 2020 followed by a sustained increase is consistent with evidence from other settings showing reduced injury-related emergency visits during the first phase of the COVID-19 pandemic, likely reflecting both changes in exposure and changes in care-seeking behavior. After 2020, however, most nonfatal injury indicators, including severe injuries, falls, and self-harm, increased in Barcelona. In contrast, road traffic injuries declined overall. Although our study does not permit causal attribution, this decline is consistent with the long-standing urban mobility and road safety policies implemented in Barcelona and with previous evaluations showing that sustained interventions were associated with substantial reductions in traffic-related injuries over time. Recent work from Barcelona estimated that more than 34,800 road traffic injuries may have been prevented between 2008 and 2023, including approximately 1000 severe injuries. These contrasting trends suggest that, although progress may have been made in road safety, other injury mechanisms are emerging as equally important or growing priorities for urban public health action [13,33].

The Barcelona system provides actionable local evidence across multiple sectors, including health, mobility, mental health, and social care. At the same time, the results make clear that improvements in data quality remain essential. In particular, the high proportion of missing external cause coding in ED data, together with the share of unspecified injury diagnoses and unknown severity, limits the precision of mechanism-specific analyses in nonadmitted cases. In addition, because the system is primarily episode-based, repeated contacts related to the same injury event may not always be distinguishable in the absence of full linkage across all sources. Regarding harmonization, linkage and the potential for double counting are essential in multisource systems. Although previous work in Barcelona demonstrated the feasibility of probabilistic linkage between police and hospital ED data for road traffic injuries, this has not yet been adopted routinely within the surveillance system, partly because of data protection constraints [34]. Future development should therefore prioritize improved external cause coding, harmonized data standards, and secure linkage capacity across health and nonhealth data sources. Recent work on digital injury surveillance platforms and data science strategies for injury prevention suggests that these steps are critical to move from fragmented routine data to robust prevention intelligence [28,35]. Several limitations should be considered when interpreting these findings. First, missing or unspecified external cause information in ED data limits mechanism-specific interpretation for nonfatal injuries treated without admission. Second, differences in coding practices, completeness, and purpose across emergency, hospitalization, mortality, and police data sources may affect comparability. Third, because the system is largely episode-based, some repeated contacts may not be fully distinguishable without individual-level linkage across all sources. Finally, although the study provides a detailed and policy-relevant picture of injury burden in Barcelona, the findings are most directly applicable to this urban context and should be generalized cautiously to settings with different health systems, coding practices, or mobility patterns.

In conclusion, this study showed that an integrated city-level injury information system can provide a substantially richer picture of injury burden than isolated routine data sources. In Barcelona, the current burden of injury is characterized by a high and increasing burden of falls among older females, an important burden of self-harm among young females, and persistently higher injury mortality among males alongside an overall decline in road traffic injuries. These findings suggest that urban injury prevention should now be framed more broadly than road safety alone and should incorporate healthy aging, fall prevention, and youth mental health as central priorities. More broadly, they support the role of integrated injury intelligence systems as essential public health tools for identifying priorities, monitoring change, and informing equitable prevention policy in cities.

## Supplementary material

10.2196/82079 Multimedia Appendix 1 Description of the variables considered in the Barcelona Injury Surveillance System (BISS).

## Abbreviations

AIS: Abbreviated Injury Score
BISS: Barcelona Injury Surveillance System
BISS-RTI: Barcelona Injury Surveillance System, specific for Road Traffic Injuries
CDC: Centers for Disease Control and Prevention
CMBD: Register of the Minimum Basic Data Set in Spain
CMBD-AH: Register of the Minimum Basic Data Set of hospital discharges
CMBD-UR: Register of the Minimum Basic Data Set of hospital emergency department visits
ED: emergency department
GUB: Register of Accidents and Victims by the Barcelona City Police
*ICD-10-CM*: *International Classification of Diseases, Tenth Revision, Clinical Modification*
MAIS: Maximum Abbreviated Injury Score
RMB: Barcelona Mortality Register
RMB-CT: Barcelona Traffic Collision Mortality Register
WHO: World Health Organization
