# Supplementary material for: The Barcelona Injury Surveillance System (BISS) for Safer Cities: Observational, Descriptive Study Using Routine Health and Police Information Databases
Source: JMIR Public Health Surveill. 2026 Jul 24;12:e82079. doi: 10.2196/82079 (PMC13399568; doi:10.2196/82079)
Supplement: Multimedia Appendix 1 [file publichealth-v12-e82079-s001.docx]

Description of the variables considered in the BISS:

- Gender: Female, Male
- Age: 0-14, 15-24, 25-44, 45-64, 65-74, 75 and older.
- Country of origin: Country according to National Institute of Statistic codes.
- Municipality of residence: Barcelona, outside Barcelona.
- Basic Health Area: (ABS for its acronym in Catalan). It is the elementary territorial unit through which primary health care services are organized (<https://www.idescat.cat/codis/?id=50&n=39&ord=1&var=2>)
- Neighborhood residence for residents in Barcelona: The city is divided territorially into 73 neighborhoods, which are grouped into 10 districts (<https://ajuntament.barcelona.cat/estadistica/catala/Territori/div84/convertidors/barris73.htm>)
- Economic financing regime: Work accident insurance, traffic collision insurance, CatSalut, and other.
- Situation of the person injured at discharge: Death, hospital admission, discharge at home, others.
- Injury diagnoses: Injury diagnoses are ICD10-CM-coded, and up to 8 diagnostic fields are available for the ED database, up to 15 fields for the HOSP database, and up to 30 fields for the MORTAL-RTI database.
- External cause: The external cause is ICD10-CM-coded, and up to 8 fields are available for the ED database and up to 5 fields for the HOSP database.
- Basic cause of death: (CBD). CBD is coded with ICD10.
- Type of injury and anatomical region affected: Each injury code in the ICD10-CM classification is further categorized using the matrix proposed by the CDC (Hedegaard et al., 2020), which classifies injuries based on the type of injury and the anatomical region affected. Using this classification system, for each injury episode, a variable is generated that indicates the presence or absence of specific injury types, as well as the involvement or non-involvement (presence of injury) of specific anatomical regions.
- Mechanism and intentionality of non-fatal injuries: The mechanism of injury and its intentionality are derived from the external cause codes in the ICD10-CM, following the CDC's classification system (Hedegaard et al., 2019). For each external cause code (codes V, W, X, Y, and T), separate variables are created to capture intentionality and the injury mechanism. Based on these, a consolidated variable is generated to indicate the presence or absence of each injury mechanism, defined by the combination of mechanism and intentionality. For external cause codes related to aggression or self-injury, the BISS categorizes intentionality as the primary injury mechanism. Conversely, for non-intentional codes, the specific mechanism is detailed. Overlap can occur in these variables when multiple external cause codes are associated with the same injury episode, as they represent various contributing mechanisms.
- Mechanism and intentionality of fatalities: Using the ICD10 codes for the external cause of the Basic Cause of Death, the CDC's classification system (NCHS, 2002) enables categorization of deaths by their intentionality and the responsible mechanism. For each external cause code, two separate variables are generated: one for intentionality and one for the mechanism. These are then combined into a single consolidated variable that captures both dimensions, providing a comprehensive understanding of the factors contributing to each death.
- User type: In the ED and HOSP databases, non-fatal traffic collision injuries under BISS-RTI are identified using the external cause V-codes for traffic collision injuries (refer to Table 3). These codes are utilized to determine the type of user involved in the collision. In contrast, the MORTAL-RTI and INJURED-RTI databases already include a dedicated variable that directly specifies the user type: Pedestrian, Passenger car users, Motorcyclist, Cyclist, Van or truck user, Bus User and Other Users.
- Severity: The overall severity of an injury is determined by analyzing all the injury diagnoses recorded for an individual. This is done using the "cat-trauma" program from the ICDPICR library version 1.0.0 in R (Clark et al., 2018). The program calculates the Abbreviated Injury Score (AIS, ranging from 1 to 6) for each valid injury code and the Maximum AIS (MAIS, also ranging from 1 to 6). Based on the MAIS, the variable MAIS3+ is created, categorizing severity as "severe" (MAIS 3–6) or "not severe" (MAIS 1–2). If no valid injury codes are available or all codes are non-specific, the three variables (AIS, MAIS, MAIS3+) are assigned the value "Unknown."

For the INJURED-RTI database, the severity classification follows police criteria. In this context, an individual involved in a traffic collision is classified as injured if they require any type of health care. The police classification further categorizes injuries as: Minor: No hospital admission required or admission lasted less than 24 hours; Serious: Hospital admission required for more than 24 hours; Fatal: Death occurring within the first 24 hours following the collision.

- Collision variables: The INJURED-RTI database, managed by the Barcelona Urban Police, includes a wide range of variables related to traffic collisions and their circumstances. These variables provide detailed contextual information, such as the type of collision, type of road, time of the incident, type of lighting, weather conditions, and the use of safety accessories by the individuals involved. This comprehensive dataset allows for a thorough analysis of the factors contributing to traffic collisions and their outcomes.

Table S1. Type of user according to the ICD10-CM codes of external cause of injury due to traffic collision.

| **Type of user** | **External cause codes ICD10-CM** |
| --- | --- |
| Pedestrian | V00.0; V01-V06.1/.9; V09.2/.3/.9 |
| Pedestrian transport users | V00.1; V00.8, except V00.81, V00.82 |
| Cyclists | V10-V18.3/.4/.5/.9; V19.4/.5/.6/.9 |
| Motorists | V20-V28.3/.4/.5/.9; V30-V38.4/.5/.6/.7/.9; V29 i V39.4/.5/.6/.9 |
| Car users | V40-V48.4/.5/.6/.7/.9; V49.4/.5/.6/.9 |
| Van or truck users | V50-V58.4/.5/.6/.7/.9; V59.4/.5/.6/.9 |
| Users of heavy transport vehicles | V60-V68.4/.5/.6/.7/.9; V69.4/.5/.6/.9 |
| Bus users | V70-V78.4/.5/.6/.7/.9; V79.4/.5/.6/.9 |
| Users of rail vehicles | V81.1; V82.1/.9 |
| Users of special vehicles (agricultural, construction, etc.) | V83-V85.0/.1/.2/.3/.4 |
| Users of another motor vehicle | V86.0/.1/.2/.3/.4 |
| Unknow | V87.0/.1/.2/.3/.4/.5/.6/.7/.8/.9; V89.2/.3/.9 |
